# Dopamine Transporter Imaging as Objective Monitoring Biomarker in Parkinson's Disease

**DOI:** 10.1002/ana.27223

**Published:** 2025-03-27

**Authors:** Verena Dzialas, Gérard N. Bischof, Kathrin Möllenhoff, Alexander Drzezga, Thilo van Eimeren

**Affiliations:** ^1^ Department of Nuclear Medicine, Faculty of Medicine, and University Hospital Cologne University of Cologne Cologne Germany; ^2^ Faculty of Mathematics and Natural Sciences University of Cologne Cologne Germany; ^3^ Molecular Organization of the Brain Research Centre Juelich, Institute for Neuroscience and Medicine II Juelich Germany; ^4^ Faculty of Medicine, Institute of Medical Statistics and Computational Biology (IMSB) University of Cologne Cologne Germany; ^5^ German Centre for Neurodegenerative Diseases Bonn Germany; ^6^ Department of Neurology, Faculty of Medicine and University Hospital Cologne University of Cologne Cologne Germany

## Abstract

**Objective:**

Although dopamine transporter (DaT) imaging is a valuable diagnostic biomarker, few studies have investigated its utility in objectively monitoring disease progression in patients with Parkinson's disease (PD). To date, no study has established a longitudinal relationship between the DaT signal decline and the motor symptom increase, potentially due to neglected factors such as brain regions, disease laterality, and symptom subtypes, which this study addresses.

**Methods:**

This cohort study included participants who met the Movement Disorder Society (MDS) criteria for PD, with longitudinal imaging and clinical data from the Parkinson's Progression Markers Initiative Database. Linear mixed model analyses were used to investigate the relationship between the DaT signal decline and the motor symptom severity increase over time. We hypothesized that a decline in putaminal DaT availability in the less affected hemisphere would be associated with increasing contralateral motor symptoms, measured by the Unified Parkinson's Disease Rating Scale (UPDRS). Additional models explored the effects of different brain regions (caudate and putamen), symptom categories (MDS UPDRSIII score with and without tremor items), and disease onset laterality (left or right hemisphere).

**Results:**

We included 719 participants (443 male patients and 276 female patients; mean age = 62.2 ± 9.5 years) with 1,981 available data points. As hypothesized, we observed a significant association between the decrease in the less affected putaminal DaT signal and motor symptom increase in the contralateral body side, independent of including or excluding tremor scores.

**Interpretation:**

Our findings support the use of repetitive DaT imaging for objectively monitoring PD progression. This could facilitate personalized disease tracking, subtyping, and intervention testing in the future. ANN NEUROL 2025;98:120–135

Dopamine transporter (DaT) single‐photon emission computed tomography (SPECT) is a well‐established, accurate tool for detecting nigro‐striatal dopaminergic neuron degeneration in Parkinson's disease (PD).^1^ Despite its established diagnostic utility in PD and observed correlations with symptom severity in cross‐sectional studies,^2^, ^3^, ^4^, ^5^ evidence for DaT imaging as an objective biomarker for monitoring disease progression is limited.^6^, ^7^, ^8^ Thus, DaT imaging is primarily used for diagnosis, whereas its potential for tracking individual disease trajectories, identifying progression subtypes, and evaluating therapies remains unclear.

In previous studies, the lack of association between DaT signal and worsening of motor symptoms is likely related to 3 main confounding factors. First, the asymmetrical pattern (ie, laterality) of dopamine neuron loss (ie, neurodegeneration) might influence the DaT signal's sensitivity to accurately depict motor symptom progression, depending on the brain region used for signal extraction. In PD, neurodegeneration typically initiates in the putamen of one hemisphere (ie, the more affected hemisphere) during preclinical stages before spreading to the ipsilateral caudate and contralateral putamen^9^, ^10^ (ie, the less affected hemisphere). Correspondingly, cross‐sectional early‐stage PD studies revealed stronger associations between DaT availability in the less affected putamen and motor symptom severity compared to DaT availability derived from the caudate nucleus or more affected putamen.^2^, ^5^ This discrepancy may be attributed to flooring effects in the primarily affected hemisphere, limiting meaningful interindividual differences.^2^, ^5^ These findings highlight the need for region‐specific analyses toward symptom progression when evaluating DaT imaging as a monitoring biomarker. Second, different aspects of movement may show varying associations with dopamine degeneration. Whereas axial and limb‐akinetic sub‐scores of the motor assessment showed significant cross‐sectional correlations with striatal DaT availability, the results for rigidity sub‐scores were inconsistent, and tremor sub‐scores showed no correlation.^2^, ^3^, ^4^, ^5^, ^8^ These findings suggest that distinct symptom categories exhibit varying degrees of association with dopamine depletion in PD. Particularly, the involvement of multiple neurotransmitter systems^11^ in tremors might obscure its association with dopamine depletion, favoring its use as a diagnostic rather than a progression marker. Third, although patients are often measured in the OFF‐medication state (ie, temporary withdraw of dopamine medication), the effects of dopaminergic medication should still be considered due to its long‐lasting benefits on symptom severity.^7^, ^12^ Additionally, certain antidepressants, like selective serotonin reuptake inhibitors, can alter DaT estimates,^13^ potentially influencing disease trajectories by masking disease progression or affecting DaT imaging.

However, these factors have only been partially addressed in longitudinal studies. Costello et al considered the most affected hemisphere,^6^ Simuni et al accounted for medication status and type,^7^ and Yang et al analyzed the sex‐ and region‐specific models.^8^ However, none of these studies found a significant association between the magnitude of DaT signal reduction and increased motor symptom severity.

Therefore, in this study, we systematically investigate the longitudinal relationship between DaT imaging and disease progression in 719 patients. We hypothesized that the putaminal DaT signal of the less affected hemisphere would most accurately depict contralateral motor impairments, particularly when excluding tremor scores. Further, we investigated how the laterality, the medication, and the brain region influence the DaT signal's ability to track motor symptom progression.

## Methods

### Data

Data used in the preparation of this article were obtained on September 22, 2024, from the Parkinson's Progression Marker Initiative (PPMI) database (https://www.ppmi-info.org/access-data-specimens/download-data), RRID:SCR_006431. For up‐to‐date information on the study, visit http://www.ppmi-info.org. Downloaded data included longitudinal DaT‐SPECT imaging and clinical data from up to 5 assessments over 5 years. Inclusion criteria consisted of: (1) a clinical diagnosis of PD according to Postuma et al^14^; (2) availability of time‐constant demographic and clinical information, including date of birth and diagnosis, sex, handedness, and years of education; and (3) at least 2 temporally separated assessment dates with DaT‐SPECT, Movement Disorder Society Unified Parkinson's Disease Rating Scale motor part III (MDS UPDRS III) in the off medication state, Montreal Cognitive Assessment (MoCA) score, levodopa equivalence daily dose (LEDD), and antidepressant medication information. Consequently, patients with only one assessment date were excluded. In accordance with the Declaration of Helsinki, ethical approval and written informed consent for all patients were obtained from the respective PPMI sites.

### Preprocessing and Variable Computation

#### Dopamine Transporter Single Photon Emission Computed Tomography

To examine how region‐specific DaT signals and laterality affect disease progression monitoring, we analyzed 9 distinct DaT signal variables as regressors. Lateral regressors comprise the signals from the more and less affected hemispheres for the caudate nucleus, putamen, and striatum (ie, combined putamen and caudate signals), whereas the non‐lateral variables represent the average across hemispheres, referred to as mean caudate, putamen, and striatum. DaT‐SPECT (123I Ioflupane ([123I]β‐CIT)) imaging followed the respective PPMI protocol (for details, see https://www.ppmi-info.org/study-design/research-documents-and-sops). Specific Binding Ratios (SBRs) from the PPMI “DaTScan_Analysis.csv” file included the left, right, and mean putamen, caudate, and striatum values. Following the PPMI protocol, SBRs were calculated using Equation 1.

(1) $$\mathrm{SBR}=\frac{\text{region of interest}}{\text{reference region}}-1\;|\;\text{reference region}=\text{occipital cortex}$$

Based on these SBRs, we established more and less affected hemispheres for each brain region and assessment date by calculating the asymmetry index (AI) by subtracting right from left SBRs and dividing the outcome by the sum of both values, as depicted in Equation 2.

(2) $$\mathrm{AI}=\frac{\left(\text{left region of interest}\;\mathrm{SBR}-\text{right region of interest}\;\mathrm{SBR}\right)}{\left(\text{left region of interest}\;\mathrm{SBR}+\text{right region of interest}\;\mathrm{SBR}\right)}$$

Negative values indicated higher DaT availability in the right hemisphere (ie, left dominant PD), whereas positive values were associated with more depletion in the right hemisphere (ie, right dominant PD). In the absence of a validated AI cutoff to identify patients with asymmetrical or symmetrical pathology, we applied a cutoff from the literature (ie, 5%)^15^ and no cutoff (ie, 0%). Accordingly, for a cutoff of AI = 0% and AI = 5%, values greater than 0 or 0.05 were classified as right‐dominant, whereas the values less than 0 or −0.05 were classified as left‐dominant PD. Values of exactly 0 or within ± 0.05 were considered symmetrical.

For asymmetrical signals, we defined more and less affected hemispheres and corresponding SBRs. No such differentiation was made for symmetrical signals, and only mean SBRs across both hemispheres were computed. Importantly, in our models, symmetrical signals were not excluded from lateral analyses (ie, models utilizing the more or less affected DaT signals as regressors). Instead, mean scores were utilized to enhance clinical applicability.

#### Clinical Scores

Like DaT signals, motor disabilities also exhibit lateralization, with the more severe symptoms typically manifesting contralateral to the more affected hemisphere.^16^, ^17^ Consequently, leveraging the DaT signal asymmetry, we defined the body side contralateral to the more affected hemisphere as “more affected,” whereas the ipsilateral body side was deemed “less affected.” We calculated the body side‐related MDS‐UPDRS‐III scores by separating limb‐akinetic‐rigid and tremor items into scores for the more and less affected side. These were then combined with non‐side‐specific axial items.

For patients without DaT signal asymmetry, we calculated a mean MDS‐UPDRS‐III score by averaging more and less affected scores along with axial items (ie, [less affected + axial score + more affected + axial score]/2). This adjustment was necessary in the lateral models to ensure that the UPDRS III scores for patients without asymmetry were comparable to those with asymmetry. Without this adjustment, the scores for patients without asymmetry would have been systematically higher than those with asymmetry. In contrast, for non‐lateral analyses, the standard MDS‐UPDRS‐III score (ie, less affected + more affected + axial items) was used.

Given that the clinically determined (higher UPDRS III score) and imaging‐determined (lower SBR value) dominant affected sides did not always align (24% for AI = 0 and 31% for AI = 5), we repeated our analyses excluding patients with non‐aligned dominant affected sides. Additionally, considering evidence suggesting tremor severity being unrelated to the dopaminergic deficit,^2^, ^4^ MDS‐UPDRS‐III scores were computed both with and without tremor items. This approach resulted in 6 motor impairment scores, comprising the more, less, and total MDS‐UPDRS‐III scores with and without tremor items, respectively.

#### Medication

To account for potential medication effects in our models, the LEDD at each scan date was computed by summing the levodopa equivalence dose of all prescribed drugs within the month of the scan. For most drugs, levodopa equivalence doses were available in the “LEDD_Concomitant_Medication_Log.csv” file of the PPMI database. These doses were calculated by multiplying the active component's milligrams (eg, 10 mg Ropinirole) by the single dose intake (eg, 2 pills), frequency (eg, 3 times daily), and conversion factor (eg, 20). Conversion factors are listed in “Methods for Calculating Levodopa Equivalent Daily Dose (LEDD) in PPMI Data” in the PPMI database. For COMT inhibitors, we first calculated the levodopa dose of medications containing levodopa and multiplied it with the appropriate inhibition factor (eg, entacapone = 0.33, tolcapone = 0.5, and opicapone = 0.5). Although Istradefylline (Nourianz) is a selective adenosine A2A receptor antagonist and not a COMT inhibitor, we treated it similarly, as suggested by Jost et al.^18^ Safinamide (xadago), zonisamide, trihexyphenidyl, and benztropine were considered with fixed levodopa doses of 150, 100, 100, and 0 mg, respectively, regardless of their frequency or dose. When LEDD calculations were not possible due to missing data (eg, unavailable prescription start dates), the LEDD was treated as missing, and the corresponding assessment date was excluded.

Given that selective serotonin reuptake inhibitors (SSRIs), a class of antidepressants, can increase striatal‐to‐nonspecific binding ratios,^13^ we also reviewed the “Concomitant_Medication_Log.csv” file for antidepressant drugs. Antidepressants were grouped into 5 categories: SSRIs, serotonin‐norepinephrine reuptake inhibitors (SNRIs), serotonin‐modulating (non‐SSRI/SNRI), other antidepressants, and no treatment. The exact search terms for each category are provided in the Supplementary Material (Methods ‐ Search terms for antidepressant medications). Like the LEDD computation, we identified medications prescribed in the month of the scan, filtered them based on our search terms, and checked for combinations of medication categories, which were rare. Accordingly, participants were assigned to a single category based on the hierarchical order outlined above (ie, SSRI, SNRI, serotonin‐modulating, other antidepressants, no treatment).

### Defining the Timeline of the Longitudinal Approach

PPMI DaT‐SPECT scans were conducted at fixed intervals, forming the basis of our timeline. To align longitudinal clinical data with this timeline, we allowed a maximum deviation of ±3 months between a scan date and the nearest clinical assessment. If multiple clinical assessments fell within this range, we selected the closest assessment to the scan date or averaged scores if equidistant. This approach ensured that all data were harmonized to a single timeline defined by the DaT SPECT availability.

The time variable was delineated by setting the time point of the first available scan (typically the baseline assessment) as the reference point (coded as 0 months). Subsequent scans were then assigned temporal intervals in the months relative to this reference. This approach enabled a more granular model fit, treating time as a continuous variable. Disease duration and age were defined as the difference between the date of the baseline scan and the date of clinical diagnosis or birth date, respectively. Because our timeline variable already captures the longitudinal information inherent in progressive age, we included only age at baseline as a time‐constant variable to prevent collinearity in the model.

### Statistical Analysis

The mixed model analyses to assess the utility of DaT‐SPECT‐derived SBRs as PD monitoring biomarkers were structured as follows. Effects of interest included SBRs of the striatum, caudate nucleus, or putamen, time, and the time*SBR interaction. Time‐varying variables, such as antidepressant medication, LEDD, and MoCA were included to address potential effects of medication and cognitive status on motor function. Moreover, time‐constant variables were added to adjust for initial differences in age, sex, handedness, years of education, and disease duration. Finally, a random intercept and slope were included, accounting for inherent data correlations due to multiple measurements per subject and allowing to model individual disease trajectories. The analysis pseudocode can be seen in Equation 3.

(3) UPDRS‐III∼Time (months, continuous)+         | Fixed effectsSBR (continuous)+                Longitudinal variablesSBR × Time (continuous)+          of interest MoCA (continuous)+Antidepressant medication (categorical)+  Longitudinal covariatesLEDD (gram, continuous)+ Age (years, continuous)+Sex (categorical)+Handedness (categorical)+           Time constant covariatesDisease duration (months, continuous)+Years of education (years, continuous)+ Residuals+Random slope (Time)+             Random effectsRandom intercept (Subject)

We initially tested our hypothesis that the putaminal DaT signal of the less affected hemisphere would most accurately depict contralateral motor impairments, excluding the tremor sub‐score. To further explore the importance of considering laterality and brain regions, we computed a total of 18 mixed models per AI cutoff. These models were based on 9 different regressors and 2 outcome variables. Mean, more, and less affected putaminal, caudate nucleus, and striatal SBRs were utilized as regressors for lateralized MDS‐UPDRS‐III scores, both with and without tremor items. Given our clearly defined hypothesis, the analysis focusing on the effect of the less affected putaminal DaT signal over time on the UPDRS III score without tremor items was not corrected for multiple comparisons. For the remaining analyses, we applied a Bonferroni correction for 8 comparisons (adjusted significance threshold = 0.05/8 ≈ 0.006). Considering that the non‐lateral analyses combine both lateral analyses (ie, more and less affected), striatal analyses combine the putamen and caudate signals, and these results are reported only for completeness and clarity, 8 unique analyses remain: more and less affected putamen and caudate on UPDRS scores, with and without tremor items. Similarly, we did not apply corrections for the different AI cutoffs, as these were included solely to demonstrate the stability of the approach and did not introduce new findings.

In addition to reporting the *p* values of all regressors, we applied the following markings to facilitate result interpretation: 0.05 > *p* > 0.006 (†) indicates results that are significant only when uncorrected; 0.006 > *p* > 0.001 (*), 0.001 > *p* > 0.0001 (**), and *p* < 0.0001 (***) indicate progressively stronger levels of significance.

In addition to the linear mixed model analyses, we extensively present the cohort's demographic, socioeconomic, and clinical factors, using means and standard deviations for continuous variables and frequencies for categorical variables. To assess potential dropout effects, the cohort was divided into completers (who underwent scanning after a 42‐month interval, completing the 4‐year imaging schedule) and non‐completers (individuals with their last scan before 42 months). For the comparison of longitudinal scores, these variables were categorized into years (year 0 = 0–6 months, year 1 = 7–18 months, year 2 = 19–30, year 3 = 31–42 months, year 4 = 43–54 months, and year 5 = 55–66) and like demographic and time‐constant variables compared between completers and non‐completers using appropriate tests such as the Mann–Whitney‐*U* test or the *χ* ^2^ test.

All analyses were performed using R^19^ version 4.2.1 and mixed models were set up using nlme (version 3.1‐157).

## Results

### Demographics

The total cohort (*n* = 719) was derived from an initial pool of 1,364 participants (see Supplementary Figure S1 for a flowchart detailing the filtering process). Participants comprised 443 male patients and 276 female patients, with an average age of 62.2 ± 9.5 years. Completers and non‐completers showed no significant differences in demographic variables, except for the follow‐up period, which by design was shorter for non‐completers (20.9 ± 6.1 months compared to 49.1 ± 2.5 months for completers). See Table 1 for demographic details.

**TABLE 1:** Demographic Characteristics of the Longitudinal PD Cohort

	Cohort Description		Group Comparison		
			‐Completers vs. Non‐Completers‐		
	Characteristics	Entire Parkinson's disease cohort	Completers	Non‐completers	*p*
			Average ± SD	Average ± SD	
Demographics	No. of patients	719	274	445	
	Age, mean (SD), yr	62.2 (9.5)	60.9 (9.5)	63.0 (9.5)	0.003
	No. (%), sex, M/F	443/276 (62/38)	168/106 (61/39)	275/170 (62/38)	0.96
	No. (%), handedness, left/**right**/mixed	69/**631**/19 (10/**88**/3)	27/**238**/9 (10/**87**/3)	42/**393**/10 (9/**88**/2)	0.68
	Disease duration, mean (SD), mo	12.5 (17.8)	12.8 (18.0)	12.3 (17.7)	0.83
	Follow‐up, mean (SD), mo	31.7 (14.6)	49.1 (2.5)	20.9 (6.1)	<1 × 10^−4^*
	Education, mean (SD), yr	16.0 (3.3)	15.7 (3.4)	16.2 (3.3)	0.03

Note: Bold formatting in rows with three categories was only used to aid visual orientation. The table presents the characteristics of the entire PD cohort, and subsets categorized as completers (individuals who underwent a scan after a 42‐month time interval) and non‐completers (individuals whose last scan was prior to 42 months). Continuous variables are expressed as mean and standard deviation (SD), while categorical variables are presented as frequencies (No.) and percentage (%, computed regarding the respective cohort or sub‐cohort). Furthermore, we compared completers and non‐completers using the Mann–Whitney *U* tests for continuous variables and the *χ* ^2^ tests for categorical variables. Corresponding *p* values are reported in the last column. The comprehensive comparison of demographics and longitudinal test scores (see Table 2) between completers and non‐completers involved 54 tests. The significance threshold was adjusted accordingly, using Bonferroni correction (alpha = 0.05/57 = 0.0009). Significant results after correction are indicated by a star (*) and gray shaded cells.

PD = Parkinson's disease.

The corresponding 1,981 data points were distributed as follows: (1) 667 at baseline (0–6 months); (2) 544 at year 1 (7–18 months); (3) 477 at year 2 (19–30 months); (4) 15 in year 3 (31–42 months); (5) 272 in year 4 (43–54 months); and (6) 6 in year 5 (55–66 months). For a comprehensive breakdown of data availability per month, see Supplementary Figure S2. To address the limited data availability in years 3 and 5, these were combined with data from years 2 and 4 for the longitudinal clinical data representation in Table 2 and the completer versus non‐completer analyses in Table 3. The comprehensive comparison across all clinical variables and time points between completers and non‐completers revealed significant differences only in the UPDRS III scores at year 0, for both AI cutoffs (p_AI_0%_ and p_AI_5%_ <0.0009, Bonferroni corrected threshold for multiple comparisons). For results based on an AI cutoff of 0%, please refer to Table 3, and for those based on an AI cutoff of 5%, refer to Supplementary Table S1.

**TABLE 2:** Longitudinal Clinical Data Using an AI Cutoff of 0%

Characteristics			Entire PD Cohort			
			Year 0	Year 1	Year 2, 3	Year > = 4
No. of patients			667	542	482	274
No. of patients with more affected putamen (left/**right**/no/**unclear**)			350/**311**/6/**0**	293/**246/**3/**0**	223/**248/**9/**2**	138/**132**/3/**1**
No. of patients with more affected caudate (left/**right**/no/**unclear**)			338/**322**/7/**0**	268/**272**/2/**0**	227/**251**/4/**0**	129/**141**/4/**0**
LEDD, mean (SD), mg			90.4 (243.0)	191.6 (243.5)	409.5 (382.0)	561.1 (364.7)
Depression medication (0/1/2/3/4)^a^			636/**21**/ 4/**5**/1/**0**	506/21/4/9/2/**0**	459/17/1/4// 0/**1**	261/**7**/1/**5**/0/**0**
MoCA, mean (SD)			26.8 (2.5)	26.7 (2.7)	26.5 (3.0)	26.5 (3.2)
MDS‐ UPDRS‐III scores mean (SD)	With tremor	Total	22.0 (10.0)	25.8 (11.0)	27.4 (11.3)	29.9 (12.6)
		More affected	17.1 (7.2)	19.5 (7.8)	20.6 (8.2)	21.6 (8.8)
		Less affected	11.6 (7.0)	14.3 (8.0)	15.4 (7.9)	17.8 (9.7)
	Without tremor	Total	17.2 (8.7)	20.2 (9.8)	22.0 (10.2)	24.3 (11.6)
		More affected	13.2 (6.1)	15.1 (6.8)	16.3 (7.2)	17.4 (7.9)
		Less affected	9.1 (6.1)	11.3 (7.0)	12.6 (7.1)	14.7 (8.7)
DaT signalmean (SD)	Putamen	Mean	0.85 (0.30)	0.75 (0.27)	0.67 (0.27)	0.57 (0.26)
		More affected	0.70 (0.27)	0.63 (0.23)	0.57 (0.25)	0.49 (0.24)
		Less affected	1.0 (0.37)	0.87 (0.33)	0.78 (0.32)	0.65 (0.28)
	Caudate nucleus	Mean	1.97 (0.55)	1.83 (0.51)	1.66 (0.53)	1.47 (0.49)
		More affected	1.79 (0.53)	1.66 (0.50)	1.50 (0.51)	1.33 (0.48)
		Less affected	2.15 (0.59)	2.00 (0.56)	1.82 (0.56)	1.61 (0.52)

Note: Bold formatting in rows with three or more categories was only used to aid visual orientation. The table presents longitudinal clinical data for the entire Parkinson's disease cohort. The more and less affected hemisphere as well as body side were determined based on an asymmetry index (AI) of 0%. Longitudinal data are summarized across 4 time points: baseline (including information from 0 to 6 months), year 1 (7 to 18 months), a combination of years 2 and 3 (19 to 42 months, due to limited year 3 data), and year 4 (beyond 42 months). Continuous variables are expressed as mean ± standard deviation (SD), whereas categorical variables are presented as frequencies (No.). The more affected body side is defined contralateral to the more affected hemisphere, the less affected body side is located ipsilateral to the more affected hemisphere).

^a^ 0 = no, 1 = selective serotonin reuptake inhibitor (SSRI), 2 = serotonin norepinephrine reuptake inhibitor (SNRI), 3 = serotonin modulating, 4 = others, 5 = ambiguity due to multiple assessment dates falling within the same time period, leading to differing categorical classifications.

AI = asymmetry index; DaT = dopamine transporter; LA = less affected; LEDD = levodopa equivalent daily dose; MDS‐UPDRS‐III = Movement Disorder Society Unified Parkinson's disease rating scale motor part III (more and less affected MDS‐UPDRS‐III scores presented in the table are based on the asymmetry in the putaminal DaT signal); MoCA = Montreal Cognitive Assessment; PD = Parkinson's disease.

**TABLE 3:** Completers vs. Non‐Completers Using an Applied AI Cutoff of 0%

Characteristics			Completers			Non‐Completers		
			Year 0	Year 1	Year 2, 3	Year 0	Year 1	Year 2, 3
No. of patients			248	186	226	419	356	256
No. of patients with more affected putamen (left/**right**/no/**unclear**)			134/**110**/4/**0**	97/**89**/0/0	107/**113**/6/**0**	216/**201**/2/**0**	196/**157**/3/**0**	116/**135**/3/**2**
No. of patients with more affected caudate (left/**right**/no/**unclear**)			120/**126**/2/**0**	88/**97**/1/**0**	107/**117/**2/**0**	218/**196**/5/**0**	180/**175**/1/**0**	120/**134**/2/**0**
LEDD, mean (SD), mg			118.0 (277.4)	174.9 (214.9)	381.7 (324.1)	74.1 (218.5)	200.4 (256.8)	434.1 (425.2)
Depression medication (0/1/2/3/4)^a^			246/**1**/0/**1**/0/**0**	181/**3**/0/**2**/0/**0**	220/**5/**0/**1**/0/**0**	390/**20**/4/**4**/1/**0**	325/**18**/4/**7**/2/**0**	239/**12**/1/**3**/0/**1**
MoCA, mean (SD)			26.5 (2.6)	26.3 (2.7)	26.1 (2.8)	26.9 (2.4)	27.0 (2.6)	26.7 (3.2)
MDS‐UPDRS‐III scores mean (SD)	With tremor	Total	19.7 (8.7)*	24.6 (10.7)	26.5 (10.8)	23.3 (10.4)*	26.4 (11.0)	28.2 (11.7)
		More affected	15.6 (6.7)*	19.2 (8.1)	20.2 (8.2)	18.0 (7.3)*	19.6 (7.7)	20.8 (8.2)
		Less affected	10.3 (6.4*)	13.2 (7.8)	14.6 (7.9)	12.4 (7.3)*	14.9 (8.0)	16.1 (7.8)
	Without tremor	Total	15.8 (7.9)	19.5 (9.9)	21.4 (10.0)	18.0 (9.1)	20.6 (9.7)	22.6 (10.4)
		More affected	12.4 (5.9)	15.0 (7.2)	16.1 (7.2)	13.7 (6.2)	15.2 (6.6)	16.4 (7.1)
		Less affected	8.3 (5.7)	10.6 (7.1)	12.1 (7.4)	9.6 (6.3)	11.6 (6.9)	13.1 (6.9)
DaT signal mean (SD)	Putamen	Mean	0.81 (0.30)	0.71 (0.25)	0.66 (0.28)	0.87 (0.30)	0.77 (0.28)	0.69 (0.27)
		More affected	0.67 (0.27)	0.60 (0.21)	0.56 (0.26)	0.71 (0.26)	0.64 (0.24)	0.59 (0.24)
		Less affected	0.95 (0.35)	0.82 (0.31)	0.76 (0.33)	1.03 (0.38)	0.90 (0.34)	0.79 (0.31)
	Caudate nucleus	Mean	1.92 (0.52)	1.81 (0.46)	1.65 (0.53)	2.00 (0.56)	1.84 (0.54)	1.66 (0.53)
		More affected	1.75 (0.50)	1.64 (0.45)	1.50 (0.52)	1.82 (0.54)	1.66 (0.52)	1.49 (0.51)
		Less affected	2.09 (0.56)	1.98 (0.51)	1.81 (0.55)	2.18 (0.61)	2.01 (0.58)	1.83 (0.57)

Note: Bold formatting in rows with three or more categories was only used to aid visual orientation. The table presents longitudinal clinical data for completers (individuals who underwent a scan after a 42‐month time interval) and non‐completers (individuals whose last scan occurred before 42 months). The more and less affected hemisphere as well as body side were determined based on an asymmetry index (AI) of 0%. Longitudinal data are summarized across four time points: baseline (including information from 0 to 6 months), year 1 (7–18 months), a combination of years 2 and 3 (19–42 months, due to limited year 3 data), and year 4 (beyond 42 months). Continuous variables are expressed as mean ± standard deviation (SD), whereas the categorical variables are presented as frequencies (No.). Completers and non‐completers were compared using Mann–Whitney *U* tests for continuous variables and *χ* ^2^ tests for categorical variables. Statistically significant differences between completers and non‐completers are indicated by shading both corresponding cells in gray and marking them with a star (*). Comparisons were only made between completers and non‐completers within the same year. In total, 57 tests were conducted to compare demographics (see Table 1) and longitudinal test scores between the 2 groups. Bonferroni correction was applied to adjust the significance threshold (alpha = 0.05/57 = 0.0009).

^a^ 0 = no, 1 = selective serotonin reuptake inhibitor (SSRI), 2 = serotonin norepinephrine reuptake inhibitor (SNRI), 3 = serotonin modulating, 4 = others, 5 = Ambiguity due to multiple assessment dates falling within the same time period, leading to differing categorical classifications.

AI = asymmetry index; LEDD = levodopa equivalent daily dose; MDS‐UPDRS‐III = Movement Disorder Society Unified Parkinson's Disease Rating Scale motor part III = (more and less affected MDS‐UPDRS‐III scores presented in the table are based on the asymmetry in the putaminal DaT signal; whereas the more affected body side is defined contralateral to the more affected hemisphere, the less affected body side is located ipsilateral to the more affected hemisphere); MoCA = Montreal Cognitive Assessment.

In the PD cohort, putaminal and caudate AI were 17% and 9% in early disease stages, reflecting lateralized neurodegeneration in the putamen and the later involvement of the caudate nucleus in the disease process. For comparison, AI values in a reference cohort of healthy controls from the PPMI database (*n* = 317) were tightly centered around 0, with an average AI of less than 5%.

### Longitudinal Analysis

**FIGURE 1:**
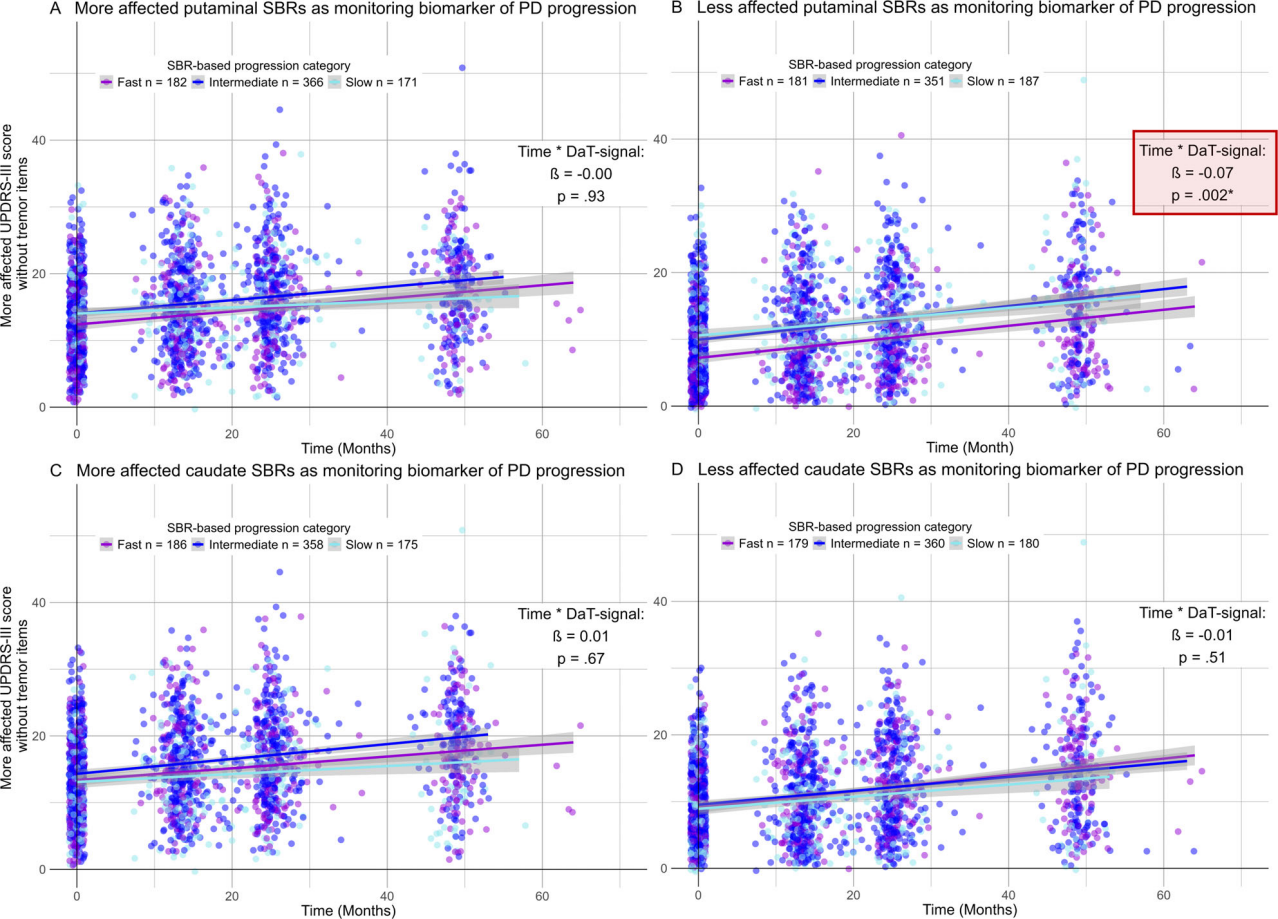
The impact of caudate and putaminal dopamine transporter decline on motor symptom progression, excluding tremor symptoms, in PD. This figure illustrates the impact of the DaT decline in PD on motor symptom progression over time, as assessed by the MDS‐UPDRS‐III score excluding tremor items. Panels (C, D) focus on the DaT signal from the caudate nucleus, whereas panels (A, B) depict the putaminal DaT signals, with distinctions made between signals from the less (B, D) and more (A, C) affected hemispheres based on an asymmetry index cutoff of 0. Each panel provides information on the *β*‐coefficient and *p* value for the corresponding interaction, offering a comprehensive view of the DaT‐dependent increase in motor symptom severity over time. As depicted, only the decline in the less affected putaminal DaT signal significantly influences the disease progression over time (*red box* in B), with patients showing faster progression in DaT signal decline also showing a steeper decline in motor symptoms. Notably, to visualize an interaction between two continuous variables in a 2D space, categorization of the DaT signal was performed. Patients were categorized into 3 groups by dividing the range of DaT signal decline over time in equal quartiles across all patients. The applied cutoff values for the caudate nucleus and putamen in the more affected and less affected hemispheres were as follows. (1) Less affected hemisphere: slow_Caudate_ > −0.095, fast_Caudate_ ≤ −0.59, slow_Putamen_ > −0.07, fast_Putamen_ ≤ −0.37; (2) More affected hemisphere: slow_Caudate_ > −0.1, fast_Caudate_ ≤ −0.5, slow_Putamen_ > −0.02, fast_Putamen_ ≤ −0.23. Moreover, a jitter of 0.5 points in height and 1 point in width was applied to prevent multiple points from overlaying each other. DaT = dopamine transporter; MDS‐UPDRS‐III score = Movement Disorder Society‐Unified Parkinson's Disease Rating Scale motor part III; PD = Parkinson's disease; SBRs = Specific Binding Rations computed as (region of interest/reference region −1), with caudate nucleus and putamen as region of interest and the occipital lobe as reference region. [Color figure can be viewed at www.annalsofneurology.org]

**FIGURE 2:**
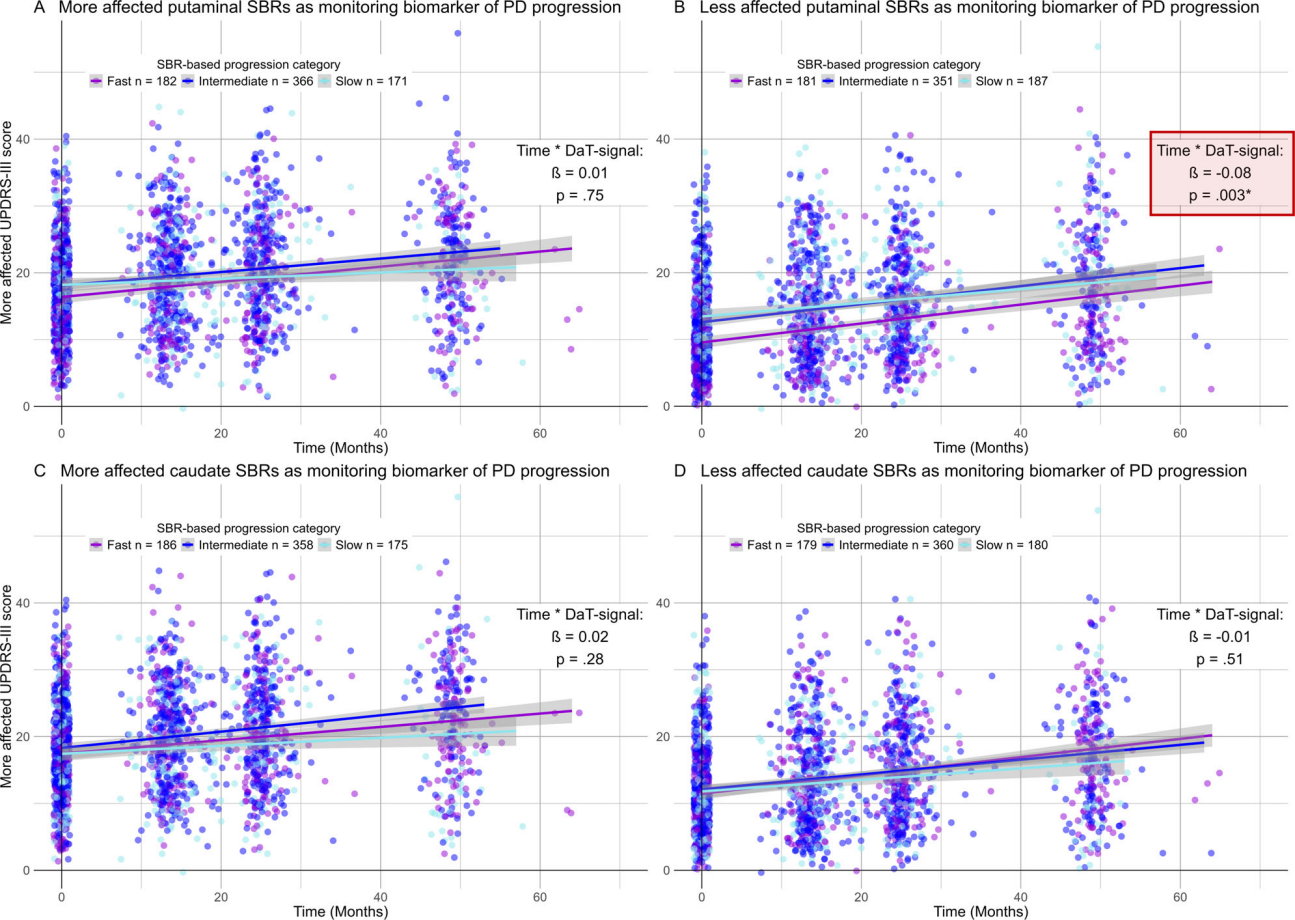
The impact of caudate and putaminal dopamine transporter decline on motor symptom progression in PD. This figure illustrates the impact of the DaT decline in PD on motor symptom progression over time, as assessed by the total MDS‐UPDRS‐III score. Panels (C, D) focus on the DaT signal from the caudate nucleus, while panels (A, B) depict the putaminal DaT signals, with distinctions made between signals from the less (B, D) and more (A, C) affected hemispheres based on an asymmetry index cutoff of 0. Each panel provides information on the *β*‐coefficient and *p* value for the corresponding interaction, offering a comprehensive view of the dopamine transporter‐dependent increase in motor symptom severity over time. As depicted, only the decline in the less affected putaminal DaT signal significantly influences the disease progression over time (*red box* in B), with patients showing faster progression in DaT signal decline also showing a steeper decline in motor symptoms. Notably, to visualize an interaction between two continuous variables in a 2D space, categorization of the DaT signal was performed. Patients were categorized into three groups by dividing the range of DaT signal decline over time in equal tertiles across all patients. The applied cutoff values for the caudate nucleus and putamen in the more affected and less affected hemispheres were as follows. (1) Less affected hemisphere: slow_Caudate_ > −0.095, fast_Caudate_ ≤ −0.59, slow_Putamen_ > −0.07, fast_Putamen_ ≤ −0.37; (2) More affected hemisphere: slow_Caudate_ > −0.1, fast_Caudate_ ≤ −0.5, slow_Putamen_ > −0.02, fast_Putamen_ ≤ −0.23. Moreover, a jitter of 0.5 points in height and 1 point in width was applied to prevent multiple points from overlaying each other. DaT = dopamine transporter; MDS‐UPDRS‐III score = Movement Disorder Society‐Unified Parkinson's Disease Rating Scale motor part III; PD = Parkinson's disease; SBRs = Specific Binding Rations computed as (region of interest/reference region −1), with caudate nucleus and putamen as region of interest and the occipital lobe as reference region. [Color figure can be viewed at www.annalsofneurology.org]

As hypothesized, only the decline in the putaminal DaT signal of the less affected hemisphere was significantly associated with the increase in contralateral MDS‐UPDRS‐III scores, both with (*β* = −0.08, *p* = 0.003) and without tremor items (*β* = −0.07, *p* = 0.002). Visualizations of these significant interactions can be found in Figures 1B and 2B. The remaining panels represent non‐significant interactions involving the less and more affected caudate nucleus (Fig 1A, C) and the more affected putamen (Fig 1D) on the UPDRS III score, both without (see Fig 1) and with tremor items included (see Fig 2). Statistical details regarding putaminal DaT signals as monitoring biomarkers of disease progression are presented in Table 4 for the MDS‐UPDRS‐III score without tremor items and in Supplementary Table S2 for the total MDS‐UPDRS‐III score.

**TABLE 4:** Capability of Putaminal DaT Signals to Monitor UPDRS‐III Score Progression Without Tremor Items (AI = 0)

Linear mixed model analysis of putaminal dopamine transporter (DaT) signal‐dependent increase in motor symptom severity without tremors						
	More affected putaminal signal		Mean putaminal signal		Less affected putaminal signal	
Predictors	*β*	*p*‐value [95% CI]	*β*	*p*‐value [95% CI]	*β*	*p*‐value [95% CI]
Intercept	14.32	<0.0001 [9.57:19.06]	17.28	<0.0001 [10.50:24.06]	6.41	0.007 [1.73:11.09]
Fixed effects (time‐dependent)						
Linear time (month)	0.09	<0.0001 [0.05:0.13]	0.18	<0.0001 [0.13:0.23]	0.15	<0.0001 [0.11:0.19]
Putaminal DaT signal	−4.87	<0.0001 [−6.30:−3.43]	−5.74	<0.0001 [−7.53:−3.95]	−3.60	<0.0001 [−4.60:−2.60]
Interaction (time*DaT signal)	−0.00	0.93 [−0.06:0.05]	−0.05	0.17 [−0.12:0.02]	−0.07	0.002 [−0.11:−0.02]
MoCA	−0.14	0.01 [−0.25:−0.03]	−0.22	0.004 [−0.38:−0.07]	−0.17	0.003 [−0.28:−0.06]
LEDD	−1.42	0.006 [−2.44:−0.41]	−1.56	0.03 [−2.99:−0.14]	−0.11	0.83 [−1.14:0.91]
Depression medication (0 = no, 1 = SSRI, 2 = SNRI, 3 = serotonin modulating, 4 = others)						
1	0.86	0.37 [−1.01:2.73]	1.77	0.19 [−0.87:4.42]	1.37	0.14 [−0.45:3.20]
2	−3.05	0.23:[−7.99:1.90]	−4.00	0.27 [−11.05:3.06]	−1.48	0.54 [−6.16:3.20]
3	−1.67	0.25 [−4.52:1.18]	−1.06	0.60 [−5.07:2.94]	−0.10	0.95 [−2.92:2.73]
4	−0.04	0.99 [−5.99:5.91]	−0.97	0.82 [−9.21:7.27]	−1.90	0.53 [−7.82:4.03]
Fixed effects (time constant)						
Age	0.06	0.004 [0.02:0.11]	0.13	=0.0001 [0.07:0.19]	0.14	<0.0001 [0.10:0.19]
Sex (0 = female, 1 = male)						
1	−0.41	0.35 [−1.28:0.45]	−0.38	0.54 [−1.60:0.85]	0.31	0.45 [−0.50:1.13]
Handedness (0 = right, 1 = left, 2 = mixed)						
1	0.48	0.50 [−0.91:1.87]	0.37	0.72 [−1.62:2.36]	0.42	0.53 [−0.89:1.73]
2	−0.10	0.94 [−2.64:2.45]	0.47	0.80 [−3.17:4.10]	1.02	0.40 [−1.38:3.41]
Disease duration	0.03	0.01 [0.0:0.06]	0.05	0.007 [0.01:0.08]	0.03	0.008 [0.01:0.06]
EDUCYR	0.14	0.03 [0.01:0.26]	0.17	0.07 [−0.01:0.35]	0.09	0.16 [−0.03:0.21]

Random effects	SD	Variance	SD	Variance	SD	Variance
Intercept (time)	4.73	22.37	6.76	45.70	4.06	16.48
Slope (time)	0.09	0.00	0.14	0.02	0.09	0.00
Residual	3.97	15.76	5.38	28.94	4.15	17.22

This table presents the results of the three linear mixed models evaluating the role of putaminal DaT signals in monitoring PD progression, as measured by the UPDRS‐III score without tremor items in the OFF‐medication state. Laterality effects were assessed using DaT signals from the more and less affected hemispheres, and mean values, based on the AI (cutoff = 0). Unstandardized *ß*‐coefficients, *p*‐values, and 95% confidence intervals (CI) for time‐dependent fixed effects are presented at the top, time‐constant covariates in the middle, and variance estimates for random effects and residuals at the bottom. Bonferroni correction for eight models (more and less affected putamen and caudate SBRs, *α* = 0.05/8 = 0.00625) was applied, with results considered significant at *p* < 0.006. Striatal SBRs and mean values were not included in the multiple comparison adjustment, as they are encompassed within the analyses of the putamen and caudate.

AI = asymmetry index; *ß* = unstandardized coefficients; CI = confidence intervals; EDUCYRS = years of education; LEDD = levodopa equivalence daily dose; MoCA = montreal cognitive assessment; SSRI = selective serotonin reuptake inhibitor; SNRI = serotonin norepinephrine reuptake inhibitor; UPDRS‐III = unified Parkinson's disease rating scale motor‐part.

Additionally, Supplementary Table S3 provides a comprehensive summary of all mixed models performed with an AI of 0%, assessing the effects of brain region and laterality by highlighting the regressors that significantly contributed to changes in symptom severity over time. Regarding the main effects, time and DaT signal were significant in all 18 linear mixed models. Whereas time exhibited a positive *β* estimate, indicating increased motor symptoms over time, disease progression was associated with lower DaT signals (ie, negative *β*‐estimates).

When considering time‐dependent covariates, MoCA scores showed negative *β* estimates that occasionally reached the corrected significance threshold but were more often significant only without correction. This may suggest a potential contribution of cognitive impairments to motor symptoms. Despite an occasional lack of significance, analysis of medication effects using LEDD scores hinted at an association between higher medication doses and reduced severity of motor symptoms. In contrast, no association between depression medication and motor symptom severity could be observed. Regarding time‐constant covariates, neither sex nor handedness contributed to the models. However, advanced age was a strong indicator of more severe symptoms. Longer disease duration and higher education, which occasionally reached the significance threshold, may also be associated with increased motor disabilities. Detailed information on the magnitude and significance of the regressors for models using putaminal, caudate nucleus, or striatal DaT signals is provided in Tables 4 and 5 and in Supplementary Tables S2 and S4 to S6. Tables 4 and 5 and Supplementary Table S4 use the MDS‐UPDRS‐III score without tremor items as the outcome variable, whereas Supplementary Tables S2, S5, and S6 use the total MDS‐UPDRS‐III score. No noteworthy differences could be observed between models using different AI cutoffs, as presented in Supplementary Tables S7 and S8 for the UPDRS‐III score without and with tremor items. Likewise excluding patients with non‐aligned more affected sides did not substantially change the results of our analyses (for details please refer to Supplementary Table S9).

**TABLE 5:** Capability of Caudate DaT Signals to Monitor UPDRS‐III Score Progression Without Tremor Items (AI = 0)

Linear mixed model analysis of caudate dopamine transporter (DaT) signal‐dependent increase in motor symptom severity without tremors						
	More affected caudate signal		Mean caudate signal		Less affected caudate signal	
Predictors	*ß*	*p*‐value [95% CI]	*ß*	*p*‐value [95% CI]	*ß*	*p*‐value [95% CI]
Intercept	16.54	<0.0001 [11.55:21.53]	16.96	<0.0001 [9.89:24.02]	4.32	0.07 [−0.37:9.02]
Fixed effects (time‐dependent)						
Linear time (month)	0.08	<0.001 [0.04:0.13]	0.15	<0.0001 [0.08:0.21]	0.12	<0.0001 [0.08:0.17]
Caudate DaT signal	−2.04	<0.0001 [−2.78:−1.29]	−2.30	<0.0001 [−3.32:−1.29]	−1.52	<0.0001 [−2.13:−0.90]
Interaction (time*DaT signal)	0.01	0.67 [−0.02:0.03]	0.00	0.80 [−0.03:0.04]	−0.01	0.51 [−0.03:0.02]
MoCA	−0.12	0.04 [−0.23:−0.01]	−0.21	0.007 [−0.37:−0.06]	−0.18	0.001 [−0.28:−0.07]
LEDD	−1.19	0.02 [−2.22:−0.17]	−1.44	<0.05 [2.87:−0.01]	−0.28	0.58 [−1.26:0.70]
Depression medication (0 = no, 1 = SSRI, 2 = SNRI, 3 = serotonin modulating, 4 = others)						
1	1.55	0.11 [−0.36:3.46]	1.97	0.15 [−0.70:4.64]	0.85	0.35 [−0.93:2.63]
2	−3.67	0.15 [−8.70:1.37]	−4.49	0.22 [−11.66:2.69]	−1.56	0.51 [−5.82:2.58]
3	−1.49	0.31 [−4.39:1.40]	−1.20	0.56 [−5.25:2.84]	−0.31	0.82 [−3.04:2.42]
4	−0.84	0.79 [−6.91:5.23]	−1.45	0.73 [−9.75:6.85]	−1.63	0.57 [−7.27:4.00]
Fixed effects (time constant)						
Age	0.04	0.09 [−0.01:0.08]	0.13	0.0001 [0.06:0.19]	0.16	<0.0001 [0.12:0.20]
Sex (0 = female, 1 = male)						
1	−0.51	0.26 [−1.40:0.38]	−0.46	0.47 [−1.72:0.80]	0.28	0.50 [−0.53:1.08]
Handedness (0 = right, 1 = left, 2 = mixed)						
1	0.37	0.61 [−1.06:1.81]	0.13	0.90 [−1.90:2.16]	0.22	0.73 [−1.07:1.52]
2	0.51	0.70 [−2.11:3.13]	0.58	0.76 [−3.12:4.29]	0.59	0.63 [−1.79:2.96]
Disease duration	0.03	0.05 [−0.00:0.05]	0.05	0.004 [0.02:0.09]	0.04	<0.001 [0.02:0.07]
EDUCYRS	0.10	0.14 [−0.03:0.23]	0.17	0.07 [−0.01:0.35]	0.13	0.03 [0.01:0.25]

Random effects	SD	Variance	SD	Variance	SD	Variance
Intercept (time)	4.87	23.72	6.94	48.16	4.20	17.64
Slope (time)	0.09	0.01	0.14	0.02	0.09	0.01
Residual	4.03	16.24	5.38	28.94	3.81	14.52

This table presents the results of the three linear mixed models evaluating the role of caudate DaT signals in monitoring PD progression, as measured by the UPDRS‐III score without tremor items in the OFF‐medication state. Laterality effects were assessed using DaT signals from the more and less affected hemispheres, and mean values, based on the AI (cutoff = 0). Unstandardized *ß*‐coefficients, *p*‐values, and 95% confidence intervals (CI) for time‐dependent fixed effects are presented at the top, time‐constant covariates in the middle, and variance estimates for random effects and residuals at the bottom. Bonferroni correction for eight models (more and less affected putamen and caudate SBRs, *α* = 0.05/8 = 0.00625) was applied, with results considered significant at *p* < 0.006. Striatal SBRs and mean values were not included in the multiple comparison adjustment, as they are encompassed within the analyses of the putamen and caudate.

AI = asymmetry index; ß = unstandardized coefficients; CI = confidence intervals; EDUCYRS = years of education; LEDD = levodopa equivalence daily dose; MoCA = montreal cognitive assessment; SSRI = selective serotonin reuptake inhibitor; SNRI = serotonin norepinephrine reuptake inhibitor; UPDRS‐III = unified Parkinson's disease rating scale motor‐part.

## Discussion

Our systematic investigation aimed to assess the utility of DaT imaging as a monitoring biomarker for disease progression in a large cohort of 719 patients with PD. Each participant underwent up to 5 DaT‐SPECT assessments during a 5‐year follow‐up period. The analysis revealed a significant association between the decline in the less affected putaminal DaT signal and the increase in motor disabilities, measured by the MDS‐UPDRS‐III score, with and without tremor items. This discovery offers novel support for using DaT imaging in clinical settings to monitor individual disease progression, differentiate progression‐based disease subtypes, and assess the efficacy of disease‐modifying therapies. However, it is noteworthy that the identified effect size was relatively small, suggesting potential limitations in the sensitivity of either the regressors (ie, DaT signal) or the outcome variable (ie, MDS‐UPDRS‐III score), as discussed below. Moreover, possible influences of confounding variables, such as medication and cognitive status, will be addressed.

**FIGURE 3:**
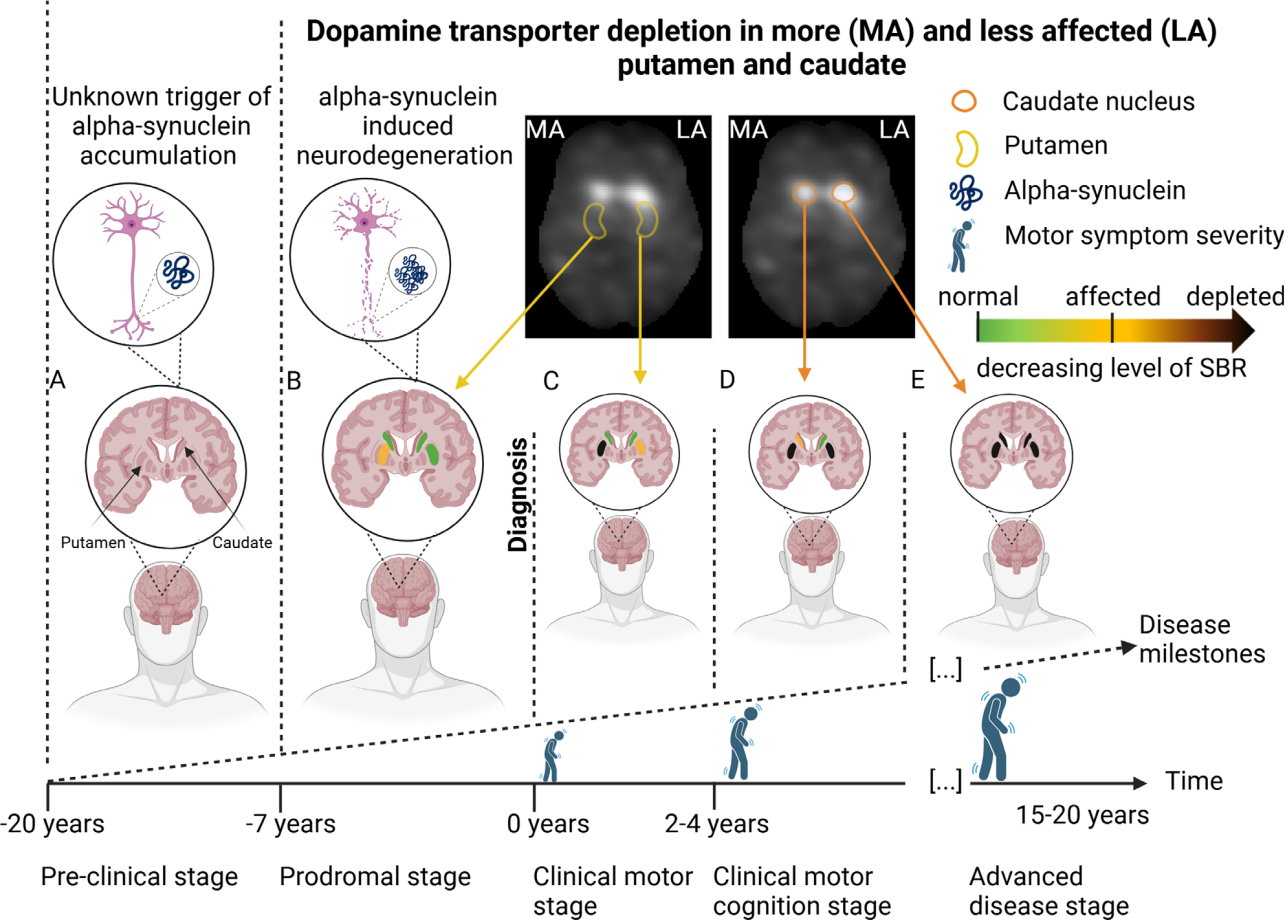
Suggested dopamine transporter‐based disease progression. The scheme illustrates the suggested timeline of PD progression, with critical timeframes displayed along the x‐axis and milestones measurable by the DaT signal represented on the z‐axis. (A) The timeline begins with the pathological accumulation of alpha‐synuclein, which is believed to start 2 decades before the clinical diagnosis.^33^ Although the underlying causes of alpha‐synuclein accumulation remain unknown, its involvement in a pathological cascade can be assumed. (B) Neurodegeneration is thought to begin approximately 7 years prior to diagnosis, primarily in the posterior putamen. Because this stage is already accompanied by diffuse non‐motor symptoms, we refer to it as the prodromal rather than preclinical stage.^34^ (C) At diagnosis, our analyses suggest that the more affected putamen is already depleted, with neurodegeneration shifting to the less affected putamen. This makes the less affected putamen a potential biomarker for monitoring disease progression during early stages. (D) The involvement of the more affected caudate nucleus then signifies a transition from motor to motor and cognitive impairments, marking intermediate disease stages. (E) Late‐stage disease is hypothesized to involve the less affected caudate. While limited DaT‐SPECT data are available for advanced disease stages, the latter part of the timeline provides a foundation for refining milestones with future research, serving as a basis for ongoing exploration rather than definitive conclusions (D, E).^9^ Importantly, individual disease trajectories might vary significantly from the proposed timeframe, based on potential disease subtypes and lifestyle factors, such as education or physical activity, that might influence compensatory mechanisms. Moreover, the illustrated linear progression might be an oversimplification of the real‐world process, serving as a starting point for further investigations into the nonlinear dynamics of clinical and imaging disease progression in certain stages. Whereas our proposed progression timeline remains speculative and requires further validation, it may provide valuable insights into disease progression. DaT = dopamine transporter; PD = Parkinson's disease; SPECT = single‐photon emission computed tomography. [Color figure can be viewed at www.annalsofneurology.org]

Our investigation into the utility of repetitive DaT imaging as a monitoring biomarker for PD progression highlighted 2 key sensitivity limitations: brain region and laterality. Given the advanced neurodegeneration in the more affected putamen at diagnosis and the later involvement of the caudate nucleus,^9^, ^10^ it is unsurprising that the effect was only evident for the less affected putaminal DaT signal. The critical role of putaminal neurodegeneration in early disease stages and its close association with symptom onset and worsening finds further support in cross‐sectional DaT‐SPECT^2^, ^4^, ^8^ and positron emission tomography (PET) studies.^16^ Moreover, regarding laterality, it was shown that lateral differences in putaminal dopamine availability correlate with left–right motor impairments, whereas no such association was found for the caudate nucleus.^16^ Combining our findings on the association between DaT signal decline and motor symptom progression with previous research, we propose the following clinical milestones. The depletion of the more affected putamen at diagnosis likely marks the transition from a prodromal to a clinical motor stage, whereas degeneration in the less affected putamen reflects early disease progression. The involvement of the more affected caudate nucleus may signify a shift to intermediate stages with cognitive decline, and late‐stage disease could be characterized by degeneration of the less affected caudate (Fig 3).

Our disease progression framework extends a recently suggested staging system, which is based on binary (±) states of alpha‐synuclein (S) accumulation and nigrostriatal dopamine degeneration (D).^20^ Although this binary framework provides a starting point, it does not account for lateral or regional variations, which are critical for understanding the nuanced dynamics of PD progression. By incorporating laterality and regional DaT signal changes, our framework allows for a more fine‐grained tracking of disease progression, identifying, for instance, the degeneration of the less affected putamen as an early biomarker of disease progression. This refinement enables more individualized and detailed disease monitoring. Considering the novelty of our proposal and the limited availability of data, the framework illustrated in Figure 3 should be regarded as an evolving and adaptable model rather than an established staging system. This adaptability allows it to incorporate future findings, particularly as more data become available on the trajectories of disease subtypes, compensatory mechanisms, and nonlinear progression trajectories.

Despite supporting the usefulness of DaT imaging as a monitoring biomarker in PD, our observed effect sizes were relatively small. However, it is crucial to interpret these results within the context of our measurement scales. The interaction term between DaT signal and time assesses whether greater dopamine neuron loss correlates with a steeper decline in motor function. Given the marginal annual increase in the MDS‐UPDRS‐III score (~2.4 points)^21^ and the much smaller decline in DaT availability,^7^ substantially larger effect sizes for the interaction within this biological framework are improbable.

Nonetheless, the large confidence intervals relative to the small effect size suggest additional noteworthy factors, limiting the sensitivity of DaT imaging when used as a monitoring biomarker. First, the extracted DaT availability originates from broad brain regions of interest, potentially overlooking subtle changes that could indicate disease progression. Second, using DaT availability from a predefined static brain region might not be sufficient to capture the dynamic disease progression over time. Particularly during transitional phases, that is, phases where the main site of pathological change may shift from one brain region or hemisphere to another, this might limit the DaT signal's utility as a monitoring biomarker. Moreover, neurodegeneration might be further advanced in some patients than in others. Therefore, the most indicative brain region might not only vary with disease progression but also between patients. Taken together, contrasting prior studies, our research affirms repetitive DaT imaging as a potentially valuable monitoring biomarker of disease progression, noting methodological decisions, like static and uniform brain region delineation, as current issues rather than intrinsic sensitivity limitations of the biomarker itself. In the next section, we will address another overlooked consideration, namely the capability of the MDS‐UPDRS‐III score, to precisely and objectively capture the subtle changes in motor performance in early disease stages.

An important consideration when assessing DaT imaging as a monitoring biomarker is recognizing that the current sensitivity restrictions are not solely attributed to the DaT signal. Although the MDS‐UPDRS‐III score is useful for categorizing disease severity (ie, 0 = normal, 1 = slight, 2 = mild, 3 = moderate, and 4 = severe),^14^ its efficacy in capturing subtle, gradual changes occurring during disease progression, especially within short temporal intervals, might be limited. Furthermore, it is essential to note that advancements from one severity category to another may not always represent an equal increase in patient impairment (eg, normal to slight **≠** moderate to severe).^22^ Inter‐rater variabilities and daily performance differences among patients are further causes for a large variance in symptom severity estimations.^22^, ^23^ Therefore, these measurement inaccuracies and restrictions may obscure the relationship between objective (ie, DaT signal) and subjective (ie, MDS‐UPDRS‐III) disease progression.

Despite the limitations inherent in the MDS‐UPDRS‐III score for capturing subtle changes in disease progression within short time intervals, as well as the inaccuracies stemming from inter‐rater variability and performance differences, there is currently no other widely accepted and applied clinical measure of disease severity. Therefore, to enhance the capability of the DaT signal to indicate disease progression, efforts should focus on minimizing sources of inaccuracies. This may entail more frequent MDS‐UPDRS‐III assessments conducted by multiple raters, enabling the averaging of scores across multiple assessments to mitigate the impact of daily performance variations and inter‐rater discrepancies. Additionally, careful consideration of differential DaT signals, laterality differences, and potential confounding factors, such as medication effects and cognitive status on motor function may further enhance the sensitivity of the approach.

Although being measured in the off‐medication state, our analysis showed that higher medication intake was associated with slower motor symptom progression. Consistent with prior research, patients on dopamine replacement therapy exhibited reduced symptom progression compared with those without medication, whereas those on other Parkinson's medications (eg, anticholinergics) showed intermediate effects.^7^ Considering that long‐term medication effects on motor function were demonstrated to persist even after drug‐specific withdrawal periods,^12^ medication represents a significant confounding factor by obscuring actual motor symptom severity.

Although current literature suggests that certain antidepressants, particularly SSRIs,^13^ can influence SBR estimates and that depression is generally associated with greater motor symptom severity,^24^, ^25^ we did not find an association between antidepressant use and disease progression. This may, however, be due to the small number of patients with depression in the cohort.

Regarding indicators of a patient's cognitive status, we observed a potential negative association between MoCA scores and motor dysfunction and a positive association with years of education. Although it has been frequently reported that cognitive abilities play a role in maintaining motor functions in PD,^26^, ^27^ the positive association with years of education might be counterintuitive. However, in Alzheimer's disease, it has been shown that patients with higher education levels can tolerate more pathology initially but then experience a faster decline in cognitive function after a certain threshold is surpassed.^28^, ^29^ Similar mechanisms have been demonstrated in PD and may help explain the observed variability in the relationship between DaT signal decline and motor symptom progression in our study.^30^ Compensatory mechanisms, whereas beneficial in counteracting the detrimental effects of neurodegeneration, could obscure this relationship by mitigating the impact of dopamine depletion on motor symptoms. In the short term, resilience mechanisms have been shown to slow down symptom progression by reducing the need for medication.^31^ However, long‐term follow‐up studies indicate a faster increase in motor symptoms once compensatory capacity is exceeded.^5^, ^32^ Although such mechanisms are likely to contribute to variability in clinical outcomes, our analysis highlights the importance of disease progression patterns and laterality in determining the utility of DaT availability as a monitoring biomarker in early PD stages.

## Limitations

Validation in more diverse clinical cohorts is needed to increase the generalizability of our findings and account for potential additional confounding factors in the relationship between the magnitude of DaT signal reduction and motor symptom severity. Whereas the PPMI database provides an exceptionally large dataset with longitudinal neuroimaging data, it primarily comprises young and mildly affected patients. However, there is a need to explore the capability of DaT availability as a monitoring biomarker across all disease stages. Additionally, our analysis was limited to investigating linear disease trajectories, given the limited number of DaT‐SPECT assessments of each patient due to radiation exposure. Despite supporting evidence suggesting a linear increase in MDS‐UPDRS‐III scores over a 5‐year follow‐up period,^21^ accounting for nonlinearity might further enhance monitoring accuracy.

## Conclusions

Contrasting prior studies, our research reinvigorates the potential of DaT imaging as a monitoring biomarker, noting methodological decisions, like static and uniform brain region delineation or random variability in the outcome measure, as current issues rather than intrinsic sensitivity limitations of the biomarker itself. Hence, our model provides a foundation for evaluating interventions targeting the recovery or preservation of dopaminergic neurons and facilitates the categorization of patients based on their unique disease trajectories.

## Author Contributions

V.D., T.v.E., A.D., and G.N.B. contributed to the conception and design of the study; V.D., G.N.B., and K.M. contributed to the acquisition and analysis of data; V.D., T.v.E., and G.N.B. contributed to drafting the text or preparing the figures.

## Potential Conflicts of Interest

A.D. reports research support and is speaker/ part of advisory Boards at/from GE Healthcare (commercial distributor of radiotracer employed and commercial vendor of imaging instrumentation). He also reports research support, stock ownership and is speaker/ part of advisory Boards at/from Siemens Healthineers (commercial vendor of imaging instrumentation).

## Supporting information


**Data S1.** Supporting Information.

## Data Availability

Deidentified participant data. How to access data: Clinical data for the PPMI cohort should be requested via the PPMI portal (https://www.ppmi-info.org/). The R script for the mixed model analyses can be requested from the first author (Verena Dzialas).
