# Supplementary material for: Dopamine Transporter Imaging as Objective Monitoring Biomarker in Parkinson's Disease
Source: Ann Neurol. 2025 Mar 27;98(1):120–35. doi: 10.1002/ana.27223 (PMC12174767; doi:10.1002/ana.27223)
Supplement: Supplementary file 1 — Data S1. Supporting Information. [file ANA-98-120-s001.docx]

**Supplementary Material**

**Methods**

**Search terms for antidepressant medications**

SSRIs = ["fluoxetine", "fluoxetin", "fluxoitine", "fluoxteine", "fluvoxetine", "fluvoxamine", "fluoxitine", "fluroxetine", "mutan", "prozac", "fluoxetine (prozac)",

"escitalopram", "escitalopram oxalate", "cipralex", "lexapro", "escitalopram (lexapro)", "escitalopram oxalate (lexapro)",

"paxil", "paxel", "paroxetine", "paraxetine", "peroxitine", "peroxetine", "paroxetine (paxil)",

"zoloft", "sertraline", "sertaline", "setraline", "sertraline (zoloft)",

"citalopram (celexa)", "citalopram",

"paroxetina",

"viibryd", "viibryd (vilazodone hcl)", "vilazodone"]

SNRIs = ["savella", "duloxetine", "duloxetene", "cymbalta ", "cymbalta (duloxetine)", "duloxetine (cymbalta)",

"venlafaxin", "venlaflaxin ", "venlafaxine", "venlaxafine", "venlaflaxine", "venlafaxina", "effexor", "efexor", "effexor (venlafaxine)",

"desvenlafaxine", "desvenafaxine", "pristiq", "desvenlafaxine (pristiq)", "desvenlafaxine succinate"]

SerotoninModulating = ["mirtazapin", "mirtazepine", "mirtazepin", "mirtazipine", "mitrazapine", "mirtazapine","mirta tad", "remeron", "mirtazapine (remeron)",

"doxepin", "docepin",

"trazodone", "trazadone", "trazadona", "trazedone",

"trintellix",

"vortioxetine", "wortioxetin","vortioxetina",

"brintalix",

"agomelatine", "agometalin", "valdoxan",

"nefazodone",

"clomipramine",

"esketamine",

"psilocybin",

"opipramol",

"ensam",

"imipramine", "imiprammine", "trimipramin",

"elatrol ", "amitriptyline", "amitryptyline", "amirtriptyline","amitriptiline", "amytriptilline", "amitryptiline",

"nortriptyline", "nortriptiline", "remergil",

"rexulti",

"buspar"]

Others = ["quetiapin", "seroquel (quetiapine)",

"bupropion", "buproprion", "wellbutrin", "welbutrin", "wellbutrin (bupropion)", "elontril",

"desipramine",

"lithium sulfate", "lithium orotate",

"craylar", "vraylar",

"auvelity"]

**Figures**

**Supplementary Figure 1**– Flowchart of exclusion criteria application


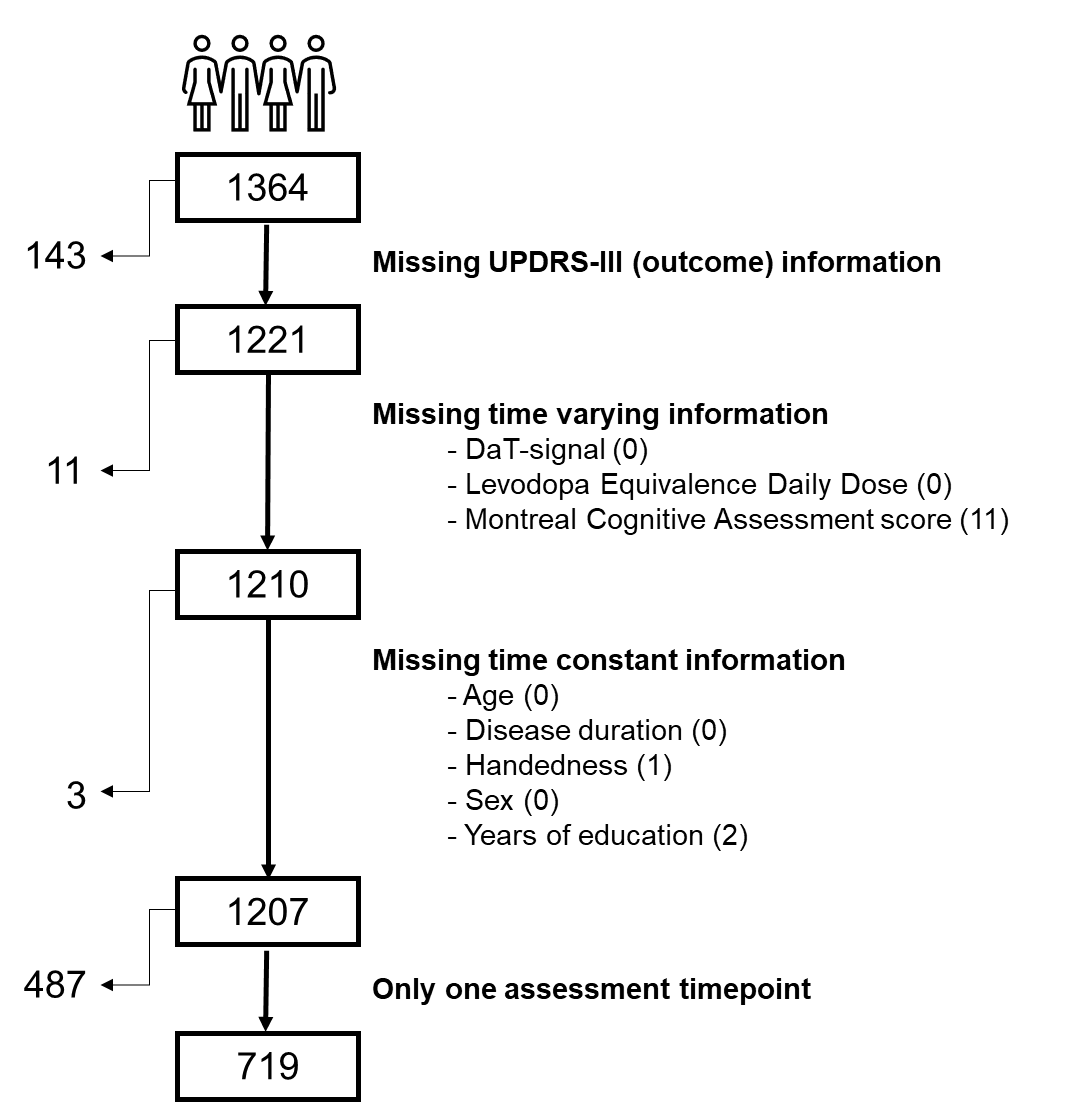


The flowchart outlines the application of exclusion criteria to 1364 patients with Parkinson’s disease, involving four filtering steps as described on the right side. The number of excluded patients at each step is shown on the left side. Most exclusions were due to unavailability of longitudinal data (n=487) or missing UPDRS-III information (n=143). Only 14 patients were excluded due to missing predictor information.

**Supplementary Figure 2** - Data availability for the linear mixed model analyses


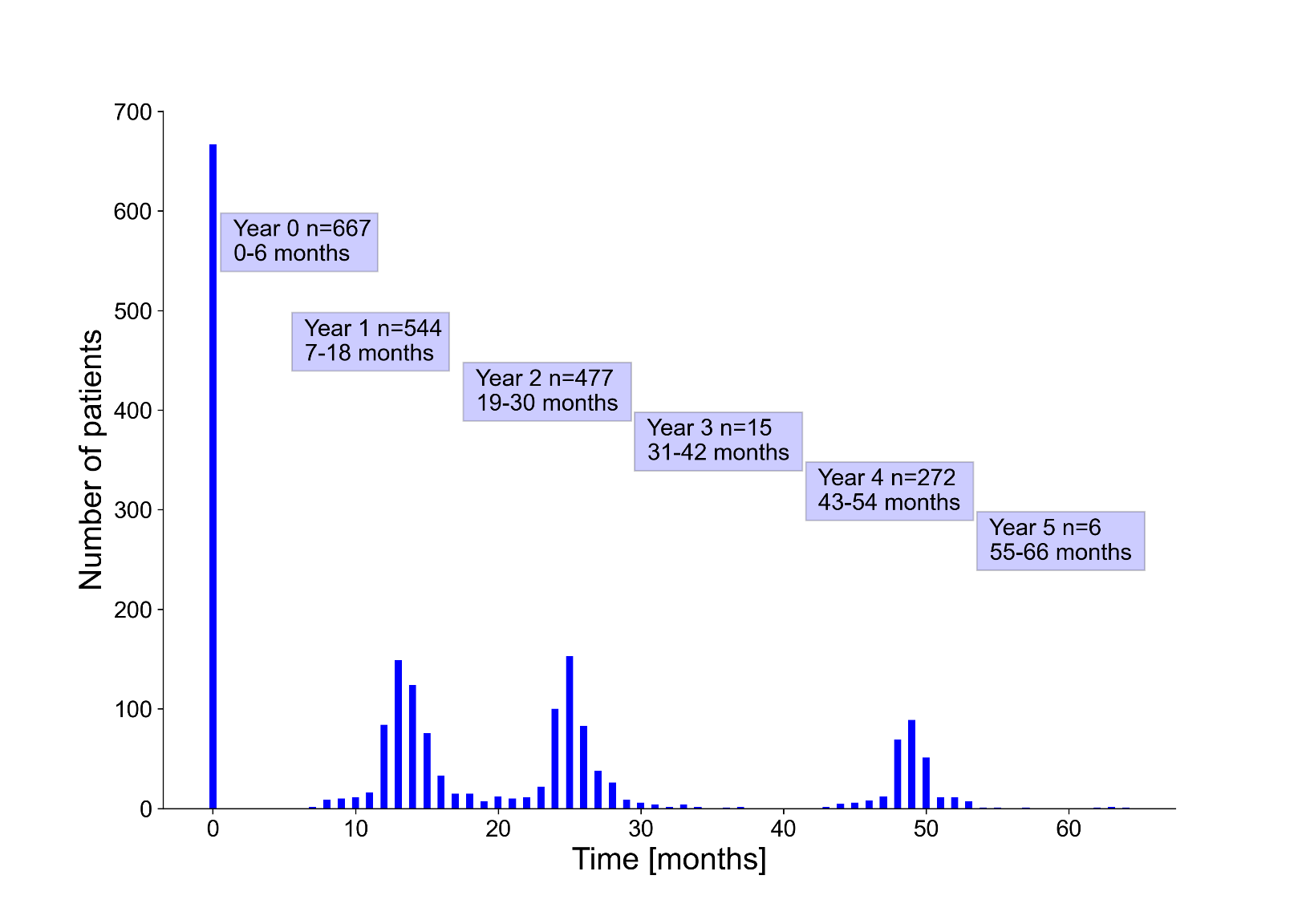


The figure displays data availability per month for 719 patients with 1981data points with longitudinal imaging and clinical information. Availability peaks at baseline and gradually decreases over time. With assessments conducted annually, peaks occur at months 12, 24, and 48, with data distribution resembling a normal curve around these peaks.

**Tables**

**Supplementary Table 1** – Longitudinal clinical data with comparison between completers vs. non-completers and an applied AI cutoff of 5%

| Cohort DEscription | | | | | | | Group comparison -Completers vs. non-Completers- | | | | | |
| --- | --- | --- | --- | --- | --- | --- | --- | --- | --- | --- | --- | --- |
| Characteristics | | | **Entire Parkinson’s disease cohort** | | | | **Completers** | | | **Non-completers** | | |
|  |  |  | **Year 0** | **Year 1** | **Year 2, 3** | **Year >= 4** | **Year 0** | **Year 1** | **Year 2,3** | **Year 0** | **Year 1** | **Year**  **2,3** |
| No. patients | | | 667 | 542 | 482 | 274 | 248 | 186 | 226 | 419 | 356 | 256 |
| No. patients with more affected putamen (left/right/no/unclear) | | | 301 / **257** / 109 / **0** | 246 / **204** / 92 / **0** | 194 / **203** / 81 / **4** | 115 / **108** / 50 / **1** | 116 / **92** / 40 / **0** | 78 / **70** / 38 / **0** | 97 / **97** / 32 / **0** | 185 / **165** / 69 / **0** | 168 / **134** / 54 / **0** | 97 / **106** / 49 / **4** |
| No. patients with more affected caudate (left/right/no/unclear) | | | 233 / **237** / 197/ **0** | 188 / **186** / 168/ **0** | 165 / **194** / 121 / **2** | 90 / **111** / 72 / **1** | 76 / **95** / 77/ **0** | 64 / **61** / 61 / / **0** | 77 / **88** / 61 / **0** | 157 / **142** / 120 / **0** | 124 / **125** / 107 / **0** | 88 / **106** / 60 / **2** |
| LEDD, mean (SD), mg | | | 90.4 (243.0) | 191.6 (243.5) | 409.5 (382.0) | 561.1 (364.7) | 118.0 (277.4) | 174.9 (214.9) | 381.7 (324.1) | 74.1 (218.5) | 200.4 (256.8) | 434.1 (425.2) |
| Depression medication (0 / 1 / 2 / 3 / 4 / 5)^1^ | | | 636 / **21**/ 4 / **5** / 1 / **0** | 506 / 21 / 4 / 9 / 2 / **0** | 459 / 17 / 1 / 4 / / 0 / **1** | 261 / **7** / 1 / **5** / 0 / **0** | 246 / **1** / 0 / **1** / 0 / **0** | 181 / **3** / 0 / **2** / 0 / **0** | 220 / **5** / 0 / **1** / 0 / **0** | 390 / **20** / 4 / **4** / 1 / **0** | 325 / **18** / 4 / **7** / 2 / **0** | 239 / **12** / 1 / **3** / 0 / **1** |
| MoCA, mean (SD) | | | 26.8 (2.5) | 26.7 (2.7) | 26.5 (3.0) | 26.5 (3.2) | 26.5 (2.6) | 26.3 (2.7) | 26.1 (2.8) | 26.9 (2.4) | 27.0 (2.6) | 26.7 (3.2) |
| MDS-UPDRS-III-scores, mean (SD) | With tremor | Total | 22.0 (10.0) | 25.8 (11.0) | 27.4 (11.3) | 29.9 (12.6) | 19.7 (8.7)* | 24.6 (10.7) | 26.5 (10.8) | 23.3 (10.4)* | 26.4 (11.0) | 28.2 (11.7) |
|  |  | More affected | 17.0 (7.1) | 19.5 (7.7) | 20.4 (8.0) | 21.5 (8.8) | 15.6 (6.6)* | 19.3 (7.8) | 20.1 (8.0) | 17.9 (7.2)* | 19.7 (7.6) | 20.7 (7.9) |
|  |  | Less affected | 11.7 (6.9) | 14.2 (7.8) | 15.6 (7.9) | 17.9 (9.4) | 10.3 (6.1)* | 13.1 (7.5) | 14.8 (7.9) | 12.5 (7.2)* | 14.8 (7.8) | 16.2 (7.9) |
|  | Without tremor | Total | 17.2 (8.7) | 20.2 (9.8) | 22.0 (10.2) | 24.3 (11.6) | 15.8 (7.9) | 19.5 (9.9) | 21.4 (10.0) | 18.0 (9.1) | 20.6 (9.7) | 22.6 (10.4) |
|  |  | More affected | 13.2 (6.0) | 15.2 (6.8) | 16.1 (7.0) | 17.3 (7.9) | 12.4 (5.8) | 15.1 (7.1) | 15.9 (7.2) | 13.7 (6.1) | 15.2 (6.6) | 16.4 (6.9) |
|  |  | Less affected | 9.2 (6.0) | 11.2 (6.8) | 12.7 (7.2) | 14.8 (8.6) | 8.3 (5.5) | 10.5 (6.9) | 12.2 (7.3) | 9.7 (6.2) | 11.6 (6.7) | 13.2 (7.0) |
| DaT Signal, mean (SD) | Putamen | Mean | 0.85 (0.30) | 0.75 (0.27) | 0.67 (0.27) | 0.57 (0.26) | 0.81 (0.30) | 0.71 (0.25) | 0.66 (0.28) | 0.87 (0.30) | 0.77 (0.28) | 0.69 (0.27) |
|  |  | More affected | 0.70 (0.27) | 0.63 (0.23) | 0.58 (0.25) | 0.49 (0.25) | 0.68 (0.27) | 0.61 (0.21) | 0.56 (0.26) | 0.72 (0.26) | 0.65 (0.24) | 0.59 (0.24) |
|  |  | Less affectd | 1.00 (0.37) | 0.87 (0.33) | 0.77 (0.32) | 0.65 (0.28) | 0.95 (0.35) | 0.82 (0.31) | 0.76 (0.32) | 1.02 (0.38) | 0.90 (0.34) | 0.79 (0.31) |
|  | Caudate nucleus | Mean | 1.97 (0.55) | 1.83 (0.51) | 1.66 (0.53) | 1.47 (0.49) | 1.92 (0.52) | 1.81 (0.46) | 1.65 (0.53) | 2.00 (0.56) | 1.84 (0.54) | 1.66 (0.53) |
|  |  | More affected | 1.79 (0.53) | 1.66 (0.50) | 1.50 (0.51) | 1.33 (0.48) | 1.76 (0.51) | 1.66 (0.46) | 1.51 (0.53) | 1.83 (0.55) | 1.68 (0.54) | 1.50 (0.52) |
|  |  | Less affected | 2.13 (0.59) | 1.99 (0.55) | 1.81 (0.56) | 1.60 (0.52) | 2.08 (0.56) | 1.97 (0.51) | 1.80 (0.55) | 2.17 (0.61) | 2.00 (0.58) | 1.82 (0.57) |

The table presents longitudinal clinical data for the entire Parkinson's disease cohort, along with subsets categorized as completers (individuals who underwent a scan after a 42-month time interval) and non-completers (individuals whose last scan occurred before 42 months). The more and less affected hemisphere as well as body side were determined based on an asymmetry index (AI) of 5%. Longitudinal data are summarized across four time points: baseline (including information from 0 to 6 months), year one (7 to 18 months), a combination of years two and three (19 to 42 months, due to limited year 3 data), and year four (beyond 42 months). Continuous variables are expressed as mean ± standard deviation (SD), while categorical variables are presented as frequencies (No.).

Completers and non-completers were compared using Mann-Whitney U tests for continuous variables and chi-square tests for categorical variables. Statistically significant differences between completers and non-completers are indicated by shading both corresponding cells in gray and marking them with a star (*). Comparisons were only made between completers and non-completers within the same year. In total, 57 tests were conducted to compare demographics (see Table 1) and longitudinal test scores between the two groups. Bonferroni correction was applied to adjust the significance threshold (alpha = 0.05 / 57 = 0.0009).

DaT = Dopamine Transporter, LA = Less Affected, LEDD = Levodopa Equivalent Daily Dose, MA = More Affected, MDS-UPDRS = Movement Disorder Society – Unified-Parkinson's-Disease-Rating-Scale (More and less affected MDS-UPDRS-III scores presented in the table are based on the asymmetry in the putaminal DaT signal. While the more affected body side is defined contralateral to the more affected hemisphere, the less affected body side is located ipsilateral to the more affected hemisphere.), MoCA = Montreal Cognitive Assessment

^1^ 0 = no , 1 = Selective Serotonin Reuptake Inhibitor (SSRI), 2 = Serotonin Norepinephrine Reuptake Inhibitor (SNRI), 3 = serotonin modulating, 4 = others, 5 = Ambiguity due to multiple assessment dates falling within the same time period, leading to differing categorical classifications

**Supplementary Table 2** - Capability of **putaminal** DaT signals to monitor UPDRS-III score progression **with tremor items** (AI = 0)

| Linear mixed model analysis of putaminal dopamine transporter signal-dependent increase in total motor symptom severity | | | | | | |
| --- | --- | --- | --- | --- | --- | --- |
|  | More affected  putaminal signal | | Mean putaminal signal | | Less affected  putaminal signal | |
| Predictors | ß | p-value  95% CI | ß | p-value  95% CI | ß | p-value  95% CI |
| Intercept | 16.48 | <.0001***  10.89:22.08 | 17.89 | <.0001***  10.16:25.62 | 5.53 | .04^†^  0.17:10.88 |
| Fixed effects (time-DEPENDENT) | | | | | | |
| Linear time (Month) | 0.11 | <.0001***  0.06:0.15 | 0.21 | <.0001***  0.14:0.27 | 0.18 | <.0001***  0.13:0.22 |
| putaminal  DaT signal | -5.24 | <.0001***  -6.96:-3.52 | -5.89 | <.0001***  -7.96:-3.82 | -3.84 | <.0001***  -5.00:-2.69 |
| Interaction  (time*DaT Signal) | 0.01 | .75  -0.06:0.08 | -0.04 | .37  -0.12:0.04 | -0.08 | .003*  -0.13:-0.03 |
| MoCA | -0.14 | .04^†^  -0.27:-0.01 | -0.22 | .01^†^  -0.40:-0.05 | -0.17 | .01^†^  -0.29:-0.04 |
| LEDD | -2.60 | <.0001***  -3.80:-1.40 | -2.66 | =.001*  -4.29:-1.04 | -0.52 | .38  -1.70:0.65 |
| Depression medication (0 = no, 1 = SSRI, 2 = SNRI, 3 = serotonin modulating, 4 = others) | | | | | | |
| 1 | 0.89 | .43  -1.31:3.09 | 1.88 | .22  -1.12:4.89 | 1.51 | .16  -0.58:3.60 |
| 2 | -2.86 | .33  -8.65:2.93 | -4.11 | .32  -12.16:3.94 | -1.83 | .50  -7.17:3.52 |
| 3 | -1.24 | .47  -4.59:2.11 | 0.23 | .92  -4.32:4.79 | 1.11 | .50  -2.12:4.34 |
| 4 | 0.66 | .85  -6.41:7.74 | -0.55 | .91  -9.95:8.85 | -1.91 | .58  -8.70:4.88 |
| Fixed effects (time constant) | | | | | | |
| Age | 0.07 | .004*  0.02:0.13 | 0.17 | <.0001***  0.10:0.24 | 0.19 | <.0001***  0.14:0.23 |
| Sex (0 = female, 1 = male) | | | | | | |
| 1 | -0.20 | .69  -1.21:0.80 | 0.13 | .86  -1.28:1.53 | 0.57 | 0.22  -0.35:1.50 |
| Handedness (0=right, 1=left, 2=mixed) | | | | | | |
| 1 | 0.22 | .79  -1.40:1.83 | 0.23 | .84  -2.03:2.49 | 0.52 | .50  -0.97:2.01 |
| 2 | -0.75 | .62  -3.71:2.20 | -0.38 | .86  -4.51:3.75 | 0.54 | .70  -2.18:3.26 |
| Disease duration | 0.03 | .08  -0.00:0.05 | 0.05 | .02^†^  0.01:0.09 | 0.03 | .03^†^  0.00:0.06 |
| Educyrs | 0.23 | .002*  0.09:0.38 | 0.28 | .007^†^  0.08:0.49 | 0.14 | .04^†^  0.00:0.28 |
| Random effects | SD | Variance | SD | Variance | SD | Variance |
| Intercept (Time) | 5.55 | 30.80 | 7.79 | 60.68 | 4.65 | 21.62 |
| Slope (time) | 0.11 | 0.01 | 0.16 | 0.03 | 0.10 | 0.01 |
| Residual | 4.80 | 23.04 | 6.15 | 37.82 | 4.79 | 22.94 |

This table presents the results of the three linear mixed models evaluating the role of **putaminal** DaT signals in monitoring PD progression, as measured by the UPDRS-III score **with tremor items** in the OFF-medication state. Laterality effects were assessed using DaT signals from the more and less affected hemispheres, and mean values, based on the AI (**cutoff=0)**. Unstandardized ß-coefficients, p-values, and 95% confidence intervals (CI) for time-dependent fixed effects are presented at the top, time-constant covariates in the middle, and variance estimates for random effects and residuals at the bottom. Bonferroni correction for eight models (more and less affected putamen and caudate SBRs, α=.05/8=.00625) was applied, with results considered significant at p<.006. Striatal SBRs and mean values were not included in the multiple comparison adjustment, as they are encompassed within the analyses of the putamen and caudate.

†<.05, *<.006, **<.001, ***<.0001 AI=Asymmetry Index, ß=unstandardized coefficients, CI=Confidence Intervals, DaT=Dopamine Transporter, EDUCYRS=years of education, LEDD=Levodopa Equivalence Daily Dose, MoCA=Montreal Cognitive Assessment, SSRI=Selective Serotonin Reuptake Inhibitor, SNRI=Serotonin Norepinephrine Reuptake Inhibitor, UPDRS-III=Unified Parkinson’s Disease Rating Scale motor-part

**Supplementary Table 3** – Comparison of results from models using the UPDRS-III score with and without tremor items as outcome (AI = 0)

| Regressors | MDS-UPDRS-III without Tremor AI = 0 | | | | | | | | | MDS-UPDRS-III AI = 0 | | | | | | | | |
| --- | --- | --- | --- | --- | --- | --- | --- | --- | --- | --- | --- | --- | --- | --- | --- | --- | --- | --- |
|  | Striatum | | | Putamen | | | Caudate | | | Striatum | | | Putamen | | | Caudate | | |
|  | MA | Mean | LA | MA | Mean | LA | MA | Mean | LA | MA | Mean | LA | MA | Mean | LA | MA | Mean | LA |
| Intercept | *** | *** | ^†^ | *** | *** | ^†^ | *** | *** |  | *** | *** |  | *** | *** | ^†^ | *** | *** |  |
| Time-dependent | | | | | | | | | | | | | | | | | | |
| Time (Month) | ** | *** | *** | *** | *** | *** | ** | *** | *** | ** | *** | *** | *** | *** | *** | ** | *** | *** |
| DaT signal | *** | *** | *** | *** | *** | *** | *** | *** | *** | *** | *** | *** | *** | *** | *** | *** | ** | *** |
| **Time*DaT** |  |  |  |  |  | ***** |  |  |  |  |  |  |  |  | ***** |  |  |  |
| MoCA | ^†^ | ^†^ | * | ^†^ | * | * | ^†^ | ^†^ | * | ^†^ | ^†^ | ^†^ | ^†^ | ^†^ | ^†^ |  | ^†^ | * |
| LEDD |  |  |  | ^†^ | ^†^ |  | ^†^ | ^†^ |  | *** | * |  | *** | * |  | ** | * |  |
| Depression medication (0 = no, 1 = SSRI, 2 = SNRI, 3 = serotonin modulating, 4 = others) | | | | | | | | | | | | | | | | | | |
| 1 |  |  |  |  |  |  |  |  |  |  |  |  |  |  |  |  |  |  |
| 2 |  |  |  |  |  |  |  |  |  |  |  |  |  |  |  |  |  |  |
| 3 |  |  |  |  |  |  |  |  |  |  |  |  |  |  |  |  |  |  |
| 4 |  |  |  |  |  |  |  |  |  |  |  |  |  |  |  |  |  |  |
| Time constant | | | | | | | | | | | | | | | | | | |
| Age | ^†^ | ** | *** | * | ** | *** |  | ** | *** | ^†^ | *** | *** | * | *** | *** |  | *** | *** |
| Sex (0 = Female, 1 = Male) | | | | | | | | | | | | | | | | | | |
| 1 |  |  |  |  |  |  |  |  |  |  |  |  |  |  |  |  |  |  |
| Handedness (1=right, 2 = left, 3= mixed) | | | | | | | | | | | | | | | | | | |
| 2 |  |  |  |  |  |  |  |  |  |  |  |  |  |  |  |  |  |  |
| 3 |  |  |  |  |  |  |  |  |  |  |  |  |  |  |  |  |  |  |
| Disease duration | ^†^ | ^†^ | * | ^†^ | ^†^ | ^†^ |  | * | ** |  | ^†^ | ^†^ |  | ^†^ | ^†^ |  | ^†^ | * |
| Years of education |  |  | ^†^ | ^†^ |  |  |  |  | ^†^ | ^†^ | ^†^ | ^†^ | * | ^†^ | ^†^ | ^†^ | ^†^ | * |

The table provides a quick overview of the results of the performed linear mixed models using an asymmetry index of 0% as cutoff to determine the more and less affected hemisphere and body side. On the left side, results for models using the MDS-UPDRS-III score without tremor items as outcome variable are shown, while the right side depicts results for the total MDS-UPDRS-III score.

DaT = Dopamine Transporter, LA = less affected, LEDD =Levodopa Equivalent Daily Dose, MA = more affected, MoCA = Montreal Cognitive Assessment, MDS-UPDRS = Movement Disorder Society - Unified Parkinson’s Disease Rating Scale

*Based on separate models for more and less affected putamen and caudate SBRs, we applied Bonferroni correction for multiple comparisons. The adjusted alpha level was set at 0.00625 (0.05/8). Therefore, results with a p-value < 0.006 were considered significant. Striatal SBRs and mean values were not included in the multiple comparison adjustment, as they are encompassed within the analyses of the putamen and caudate.* ^†^*<.05, *<.006, **<0.001, ***<.0001*

**Supplementary Table 4** - Capability of **striatal** DaT signals to monitor UPDRS-III score progression **without tremor items** (AI = 0)

| Linear mixed model analysis of Striatal dopamine transporter signal-dependent increase in motor symptom severity WITHOUT TREMORS | | | | | | |
| --- | --- | --- | --- | --- | --- | --- |
|  | More affected  striatal signal | | Mean striatal signal | | Less affected  striatal signal | |
| Predictors | ß | p-value  95% CI | ß | p-value  95% CI | ß | p-value  95% CI |
| Intercept | 16.89 | <.0001***  12.03:21.76 | 18.22 | <.0001***  11.22:25.22 | 5.26 | .03^†^  0.60:9.92 |
| Fixed effects (time-DEPENDENT) | | | | | | |
| Linear time (Month) | 0.09 | =.0001**  0.04:0.13 | 0.16 | <.0001***  0.09:0.22 | 0.13 | <.0001***  0.09:0.17 |
| striatal  DaT signal | -3.36 | <.0001***  -4.40:-2.31 | -3.94 | <.0001***  -5.33:-2.55 | -2.41 | <.0001***  -3.22:-1.60 |
| Interaction  (time*DaT Signal) | 0.01 | .74  -0.03:0.04 | -0.01 | .71  -0.06:0.04 | -0.03 | .11  -0.06:0.01 |
| MoCA | -0.14 | .01^†^  -0.25:-0.03 | -0.22 | .006^†^  -0.37:-0.06 | -0.16 | .003*  -0.27:-0.06 |
| LEDD | -1.53 | .003*  -2.53:-0.52 | -1.54 | .03^†^  -2.97:-0.11 | -0.14 | .78  -1.11:0.83 |
| Depression medication (0 = no, 1 = SSRI, 2 = SNRI, 3 = serotonin modulating, 4 = others) | | | | | | |
| 1 | 1.20 | .21  -0.67:3.07 | 1.87 | .17  -0.79:4.52 | 1.04 | .25  -0.72:2.81 |
| 2 | -3.69 | .14  -8.64:1.27 | -4.22 | .24  -11.33:2.89 | -1.24 | .60  -5.85:3.37 |
| 3 | -1.84 | .20  -4.68:1.00 | -1.09 | .59  -5.12:2.93 | 0.14 | .92  -2.57:2.85 |
| 4 | -0.67 | .83  -6.60:5.26 | -1.38 | .74  -9.66:6.90 | -1.43 | .62  -7.05:4.19 |
| Fixed effects (time constant) | | | | | | |
| Age | 0.05 | .03^†^  0.01:0.09 | 0.12 | <.001**  0.06:0.19 | 0.15 | <.0001***  0.11:0.19 |
| Sex (0 = female, 1 = male) | | | | | | |
| 1 | -0.50 | .26  -1.38:0.37 | -0.48 | .45  -1.73:0.77 | 0.26 | .52  -0.54:1.07 |
| Handedness (0=right, 1=left, 2=mixed) | | | | | | |
| 1 | 0.53 | .46  -0.88:1.94 | 0.24 | .82  -1.77:2.25 | 0.17 | .80  -1.13:1.47 |
| 2 | 0.31 | .82  -2.27:2.88 | 0.53 | .78  -3.14:4.20 | 0.72 | .55  -1.64:3.09 |
| Disease duration | 0.03 | <.05^†^  0.00:0.05 | 0.05 | .007^†^  0.01:0.08 | 0.04 | =.001*  0.01:0.06 |
| Educyrs | 0.11 | 0.11  -0.02:0.23 | 0.17 | .06  -0.01:0.36 | 0.13 | .04^†^  0.01:0.24 |
| Random effects | SD | Variance | SD | Variance | SD | Variance |
| Intercept (Time) | 4.77 | 22.75 | 6.85 | 46.92 | 4.19 | 17.56 |
| Slope (time) | 0.09 | 0.01 | 0.14 | 0.02 | 0.09 | 0.01 |
| Residual | 3.92 | 15.37 | 5.38 | 28.94 | 3.80 | 14.44 |

This table presents the results of the three linear mixed models evaluating the role of **striatal** DaT signals in monitoring PD progression, as measured by the UPDRS-III score **without tremor items** in the OFF-medication state. Laterality effects were assessed using DaT signals from the more and less affected hemispheres, and mean values, based on the AI (**cutoff=0)**. Unstandardized ß-coefficients, p-values, and 95% confidence intervals (CI) for time-dependent fixed effects are presented at the top, time-constant covariates in the middle, and variance estimates for random effects and residuals at the bottom. Bonferroni correction for eight models (more and less affected putamen and caudate SBRs, α=.05/8=.00625) was applied, with results considered significant at p<.006. Striatal SBRs and mean values were not included in the multiple comparison adjustment, as they are encompassed within the analyses of the putamen and caudate.

†<.05, *<.006, **<.001, ***<.0001 AI=Asymmetry Index, ß=unstandardized coefficients, CI=Confidence Intervals, DaT=Dopamine Transporter, EDUCYRS=years of education, LEDD=Levodopa Equivalence Daily Dose, MoCA=Montreal Cognitive Assessment, SSRI=Selective Serotonin Reuptake Inhibitor, SNRI=Serotonin Norepinephrine Reuptake Inhibitor, UPDRS-III=Unified Parkinson’s Disease Rating Scale motor-part

**Supplementary Table 5** - Capability of **caudate** DaT signals to monitor UPDRS-III score progression **with tremor items** (AI = 0)

| Linear mixed model analysis of caudate dopamine transporter signal-dependent increase in  total motor symptom severity | | | | | | |
| --- | --- | --- | --- | --- | --- | --- |
|  | More affected  caudate signal | | Mean caudate signal | | Less affected  caudate signal | |
| Predictors | ß | p-value  95% CI | ß | p-value  95% CI | ß | p-value  95% CI |
| Intercept | 18.14 | <.0001***  12.28:24.00 | 16.93 | <.0001***  8.88:24.99 | 3.15 | .25  -2.22:8.52 |
| Fixed effects (time-DEPENDENT) | | | | | | |
| Linear time (Month) | 0.09 | <.001**  0.04:0.15 | 0.16 | <.0001***  0.09:0.24 | 0.14 | <.0001***  0.09:0.19 |
| caudate  DaT signal | -1.94 | <.0001***  -2.83:-1.05 | -2.15 | <.001**  -3.31:-0.98 | -1.56 | <.0001***  -2.28:-0.85 |
| Interaction  (time*DaT Signal) | 0.02 | .28  -0.01:0.05 | 0.02 | .39  -0.02:0.06 | -0.01 | .51  -0.04:0.02 |
| MoCA | -0.11 | .11  -0.24:0.02 | -0.21 | .02^†^  -0.39:-0.04 | -0.18 | .004*  -0.30:-0.06 |
| LEDD | -2.18 | <.001**  -3.40:-0.96 | -2.49 | .003*  -4.12:-0.86 | -0.77 | .18  -1.89:0.35 |
| Depression medication (0 = no, 1 = SSRI, 2 = SNRI, 3 = serotonin modulating, 4 = others) | | | | | | |
| 1 | 1.73 | .13  -0.51:3.97 | 2.12 | .17  -0.92:5.16 | 0.90 | .38  -1.13:2.92 |
| 2 | -9.55 | .22  -7.88:2.24 | -4.73 | .26  -12.90:3.44 | -1.94 | .47  -7.21:3.34 |
| 3 | -1.47 | .40  -4.88:1.93 | 0.04 | .99  -4.56:4.64 | 1.26 | .43  -1.85:4.37 |
| 4 | -0.13 | .97  -7.30:7.04 | -1.02 | .83  -10.48:8.43 | -1.63 | .62  -8.10:4.84 |
| Fixed effects (time constant) | | | | | | |
| Age | 0.05 | .06  -0.00:0.10 | 0.17 | <.0001***  0.09:0.24 | 0.20 | <.0001***  0.16:0.25 |
| Sex (0 = female, 1 = male) | | | | | | |
| 1 | -0.24 | .64  -1.28:0.79 | 0.07 | .92  -1.36:1.50 | 0.50 | .29  -0.42:1.42 |
| Handedness (0=right, 1=left, 2=mixed) | | | | | | |
| 1 | 0.28 | .74  -1.38:1.94 | -0.04 | .97  -2.34:2.26 | 0.10 | .89  -1.37:1.58 |
| 2 | -0.12 | .94  -3.15:2.92 | -0.28 | .90  -4.48:3.93 | 0.03 | .98  -2.67:2.72 |
| Disease duration | 0.02 | .13  -0.01:0.05 | 0.05 | .01^†^  0.01:0.09 | 0.04 | .003*  0.01:0.07 |
| Educyrs | 0.17 | .03^†^  0.02:0.32 | 0.28 | .009^†^  0.07:0.49 | 0.20 | .003*  0.07:0.34 |
| Random effects | SD | Variance | SD | Variance | SD | Variance |
| Intercept (Time) | 5.66 | 32.04 | 7.96 | 63.36 | 4.82 | 23.23 |
| Slope (time) | 0.11 | 0.01 | 0.16 | 0.03 | 0.10 | 0.01 |
| Residual | 4.83 | 23.33 | 6.13 | 37.58 | 4.41 | 19.45 |

This table presents the results of the three linear mixed models evaluating the role of **caudate** DaT signals in monitoring PD progression, as measured by the UPDRS-III score **with tremor items** in the OFF-medication state. Laterality effects were assessed using DaT signals from the more and less affected hemispheres, and mean values, based on the AI (**cutoff=0)**. Unstandardized ß-coefficients, p-values, and 95% confidence intervals (CI) for time-dependent fixed effects are presented at the top, time-constant covariates in the middle, and variance estimates for random effects and residuals at the bottom. Bonferroni correction for eight models (more and less affected putamen and caudate SBRs, α=.05/8=.00625) was applied, with results considered significant at p<.006. Striatal SBRs and mean values were not included in the multiple comparison adjustment, as they are encompassed within the analyses of the putamen and caudate.

†<.05, *<.006, **<.001, ***<.0001 AI=Asymmetry Index, ß=unstandardized coefficients, CI=Confidence Intervals, DaT=Dopamine Transporter, EDUCYRS=years of education, LEDD=Levodopa Equivalence Daily Dose, MoCA=Montreal Cognitive Assessment, SSRI=Selective Serotonin Reuptake Inhibitor, SNRI=Serotonin Norepinephrine Reuptake Inhibitor, UPDRS-III=Unified Parkinson’s Disease Rating Scale motor-part

**Supplementary Table 6** - Capability of **striatal** DaT signals to UPDRS-III score progression **with tremor items** (AI = 0)

| Linear mixed model analysis of Striatal dopamine transporter signal-dependent increase in total motor symptom severity | | | | | | |
| --- | --- | --- | --- | --- | --- | --- |
|  | More affected  striatal signal | | Mean striatal signal | | Less affected  striatal signal | |
| Predictors | ß | p-value  95% CI | ß | p-value  95% CI | ß | p-value  95% CI |
| Intercept | 18.88 | <.0001***  13.17:24.59 | 18.38 | <.0001***  10.39:26.37 | 3.76 | .16  -1.55:9.07 |
| Fixed effects (time-DEPENDENT) | | | | | | |
| Linear time (Month) | 0.10 | =.0001**  0.05:0.15 | 0.18 | <.0001***  0.10:0.25 | 0.15 | <.0001***  0.10:0.20 |
| striatal  DaT signal | -3.32 | <.0001***  -4.56:-2.08 | -3.81 | <.0001***  -5.41:-2.21 | -2.46 | <.0001***  -3.39:-1.53 |
| Interaction  (time*DaT Signal) | 0.02 | .40  -0.03:0.07 | 0.01 | .79  -0.05:0.07 | -0.03 | .14  -0.06:0.01 |
| MoCA | -0.14 | .03^†^  -0.27:-0.01 | -0.21 | .02^†^  -0.39:-0.04 | -0.15 | .01^†^  -0.27:-0.03 |
| LEDD | -2.60 | <.0001***  -3.78:-1.41 | -2.60 | .002*  -4.23:-0.98 | -0.60 | .29  -1.70:0.50 |
| Depression medication (0 = no, 1 = SSRI, 2 = SNRI, 3 = serotonin modulating, 4 = others) | | | | | | |
| 1 | 1.3 | .25  -0.90:3.49 | 2.00 | .19  -1.02:5.03 | 1.17 | .25  -0.84:3.18 |
| 2 | -3.74 | .21  -9.53:2.05 | -4.43 | .29  -12.55:3.70 | -1.58 | .55  -6.81:3.65 |
| 3 | -1.65 | .33  -4.98:2.05 | 0.16 | .94  -4.42:4.75 | 1.58 | .31  -1.49:4.65 |
| 4 | 0.01 | 1  -6.98:7.00 | -0.95 | .84  -10.38:8.49 | -1.28 | .70  -7.68:5.13 |
| Fixed effects (time constant) | | | | | | |
| Age | 0.06 | .02^†^  0.00:0.11 | 0.16 | <.0001***  0.09:0.24 | 0.19 | <.0001***  0.14:0.24 |
| Sex (0 = female, 1 = male) | | | | | | |
| 1 | -0.32 | .54  -1.24:0.78 | 0.04 | .95  -1.38:1.46 | 0.54 | .24  -0.37:1.46 |
| Handedness (0=right, 1=left, 2=mixed) | | | | | | |
| 1 | 0.35 | .67  -1.37:1.88 | 0.07 | .95  -2.21:2.35 | 0.14 | .85  -1.33:1.61 |
| 2 | -0.41 | .79  -3.66:2.28 | -0.32 | .88  -4.50:3.85 | 0.25 | .86  -2.44:2.93 |
| Disease duration | 0.02 | .15  -0.01:0.05 | 0.05 | .02^†^  0.01:0.09 | 0.04 | .01^†^  0.01:0.06 |
| Educyrs | 0.19 | .01^†^  0.04:0.34 | 0.28 | .008^†^  0.08:0.49 | 0.19 | .006^†^  0.05:0.32 |
| Random effects | SD | Variance | SD | Variance | SD | Variance |
| Intercept (Time) | 5.56 | 30.91 | 7.89 | 62.25 | 4.80 | 23.04 |
| Slope (time) | 0.10 | 0.01 | 0.16 | 0.03 | 0.10 | 0.01 |
| Residual | 4.68 | 21.90 | 6.14 | 37.70 | 4.34 | 18.84 |

This table presents the results of the three linear mixed models evaluating the role of **striatal** DaT signals in monitoring PD progression, as measured by the UPDRS-III score **with tremor items** in the OFF-medication state. Laterality effects were assessed using DaT signals from the more and less affected hemispheres, and mean values, based on the AI (**cutoff=0)**. Unstandardized ß-coefficients, p-values, and 95% confidence intervals (CI) for time-dependent fixed effects are presented at the top, time-constant covariates in the middle, and variance estimates for random effects and residuals at the bottom. Bonferroni correction for eight models (more and less affected putamen and caudate SBRs, α=.05/8=.00625) was applied, with results considered significant at p<.006. Striatal SBRs and mean values were not included in the multiple comparison adjustment, as they are encompassed within the analyses of the putamen and caudate.

†<.05, *<.006, **<.001, ***<.0001 AI=Asymmetry Index, ß=unstandardized coefficients, CI=Confidence Intervals, DaT=Dopamine Transporter, EDUCYRS=years of education, LEDD=Levodopa Equivalence Daily Dose, MoCA=Montreal Cognitive Assessment, SSRI=Selective Serotonin Reuptake Inhibitor, SNRI=Serotonin Norepinephrine Reuptake Inhibitor, UPDRS-III=Unified Parkinson’s Disease Rating Scale motor-part

**Supplementary Table 7** - Comparison of results from models using the UPDRS-III score without tremor items as the outcome with different AI cutoffs: 0% and 5%

| Regressors | MDS-UPDRS-III without tremor AI = 0 | | | | | | | | | MDS-UPDRS-III without tremor AI = 5 | | | | | | | | |
| --- | --- | --- | --- | --- | --- | --- | --- | --- | --- | --- | --- | --- | --- | --- | --- | --- | --- | --- |
|  | Striatum | | | Putamen | | | Caudate | | | Striatum | | | Putamen | | | Caudate | | |
|  | MA | Mean | LA | MA | Mean | LA | MA | Mean | LA | MA | Mean | LA | MA | Mean | LA | MA | Mean | LA |
| Intercept | *** | *** | ^†^ | *** | *** | ^†^ | *** | *** |  | *** | *** | ^†^ | *** | *** | ^†^ | *** | *** | ^†^ |
| Time-dependent | | | | | | | | | | | | | | | | | | |
| Time (Month) | ** | *** | *** | *** | *** | *** | ** | *** | *** | ** | *** | *** | *** | *** | *** | ** | *** | *** |
| DaT signal | *** | *** | *** | *** | *** | *** | *** | *** | *** | *** | *** | *** | *** | *** | *** | *** | *** | *** |
| **Time*DaT** |  |  |  |  |  | ***** |  |  |  |  |  |  |  |  | ***** |  |  |  |
| MoCA | ^†^ | ^†^ | * | ^†^ | * | * | ^†^ | ^†^ | * | * | ^†^ | ^†^ | * | * | ^†^ | ^†^ | ^†^ | * |
| LEDD |  |  |  | ^†^ | ^†^ |  | ^†^ | ^†^ |  | ^†^ |  |  | * | ^†^ |  | ^†^ | ^†^ |  |
| Depression medication (0 = no, 1 = SSRI, 2 = SNRI, 3 = serotonin modulating, 4 = others) | | | | | | | | | | | | | | | | | | |
| 1 |  |  |  |  |  |  |  |  |  |  |  |  |  |  |  |  |  |  |
| 2 |  |  |  |  |  |  |  |  |  |  |  |  |  |  |  |  |  |  |
| 3 |  |  |  |  |  |  |  |  |  |  |  |  |  |  |  |  |  |  |
| 4 |  |  |  |  |  |  |  |  |  |  |  |  |  |  |  |  |  |  |
| Time constant | | | | | | | | | | | | | | | | | | |
| Age | ^†^ | ** | *** | * | ** | *** |  | ** | *** |  | ** | *** | * | ** | *** |  | ** | *** |
| Sex (0 = Female, 1 = Male) | | | | | | | | | | | | | | | | | | |
| 1 |  |  |  |  |  |  |  |  |  |  |  |  |  |  |  |  |  |  |
| Handedness (1=right, 2 = left, 3= mixed) | | | | | | | | | | | | | | | | | | |
| 2 |  |  |  |  |  |  |  |  |  |  |  |  |  |  |  |  |  |  |
| 3 |  |  |  |  |  |  |  |  |  |  |  |  |  |  |  |  |  |  |
| Disease duration | ^†^ | ^†^ | * | ^†^ | ^†^ | ^†^ |  | * | ** |  | ^†^ | * | ^†^ | ^†^ | ^†^ |  | * | ** |
| Years of education |  |  | ^†^ | ^†^ |  |  |  |  | ^†^ | ^†^ |  |  | ^†^ |  |  |  |  | ^†^ |

The table provides a quick overview of the results of the performed linear mixed models using the UPDRS-III score without tremor items as outcome. On the left side, results for models using an asymmetry index (AI) of 0% as cutoff are shown, while the right side depicts results for an asymmetry index of 5%.

DaT = Dopamine Transporter, LA = less affected, LEDD =Levodopa Equivalent Daily Dose, MA = more affected, MoCA = Montreal Cognitive Assessment, MDS-UPDRS = Movement Disorder Society - Unified Parkinson’s Disease Rating Scale

*Based on separate models for more and less affected putamen and caudate SBRs, we applied Bonferroni correction for multiple comparisons. The adjusted alpha level was set at 0.00625 (0.05/8). Therefore, results with a p-value < 0.006 were considered significant. Striatal SBRs and mean values were not included in the multiple comparison adjustment, as they are encompassed within the analyses of the putamen and caudate.* ^†^*<.05, *<.006, **<0.001, ***<.0001*

**Supplementary Table 8** - Comparison of results from models using the UPDRS-III score as the outcome with different AI cutoffs: 0% and 5%

| Regressors | MDS-UPDRS-III AI = 0 | | | | | | | | | MDS-UPDRS-III AI = 5 | | | | | | | | |
| --- | --- | --- | --- | --- | --- | --- | --- | --- | --- | --- | --- | --- | --- | --- | --- | --- | --- | --- |
|  | Striatum | | | Putamen | | | Caudate | | | Striatum | | | Putamen | | | Caudate | | |
|  | MA | Mean | LA | MA | Mean | LA | MA | Mean | LA | MA | Mean | LA | MA | Mean | LA | MA | Mean | LA |
| Intercept | *** | *** |  | *** | *** | ^†^ | *** | *** |  | *** | *** |  | *** | *** |  | *** | *** |  |
| Time-dependent | | | | | | | | | | | | | | | | | | |
| Time (Month) | ** | *** | *** | *** | *** | *** | ** | *** | *** | ** | *** | *** | *** | *** | *** | ** | *** | *** |
| DaT signal | *** | *** | *** | *** | *** | *** | *** | ** | *** | *** | *** | *** | *** | *** | *** | *** | ** | *** |
| **Time*DaT** |  |  |  |  |  | ***** |  |  |  |  |  |  |  |  | ***** |  |  |  |
| MoCA | ^†^ | ^†^ | ^†^ | ^†^ | ^†^ | ^†^ |  | ^†^ | * | ^†^ | ^†^ | ^†^ | ^†^ | ^†^ | ^†^ | ^†^ | ^†^ | ^†^ |
| LEDD | *** | * |  | *** | * |  | ** | * |  | ** | * |  | *** | * |  | ** | * |  |
| Depression medication (0 = no, 1 = SSRI, 2 = SNRI, 3 = serotonin modulating, 4 = others) | | | | | | | | | | | | | | | | | | |
| 1 |  |  |  |  |  |  |  |  |  |  |  |  |  |  |  |  |  |  |
| 2 |  |  |  |  |  |  |  |  |  |  |  |  |  |  |  |  |  |  |
| 3 |  |  |  |  |  |  |  |  |  |  |  |  |  |  |  |  |  |  |
| 4 |  |  |  |  |  |  |  |  |  |  |  |  |  |  |  |  |  |  |
| Time constant | | | | | | | | | | | | | | | | | | |
| Age | ^†^ | *** | *** | * | *** | *** |  | *** | *** | ^†^ | *** | *** | * | *** | *** | ^†^ | *** | *** |
| Sex (0 = Female, 1 = Male) | | | | | | | | | | | | | | | | | | |
| 1 |  |  |  |  |  |  |  |  |  |  |  |  |  |  |  |  |  |  |
| Handedness (1=right, 2 = left, 3= mixed) | | | | | | | | | | | | | | | | | | |
| 2 |  |  |  |  |  |  |  |  |  |  |  |  |  |  |  |  |  |  |
| 3 |  |  |  |  |  |  |  |  |  |  |  |  |  |  |  |  |  |  |
| Disease duration |  | ^†^ | ^†^ |  | ^†^ | ^†^ |  | ^†^ | * |  | ^†^ | ^†^ |  | ^†^ | ^†^ |  | ^†^ | * |
| Years of education | ^†^ | ^†^ | ^†^ | * | ^†^ | ^†^ | ^†^ | ^†^ | * | * | ^†^ | ^†^ | * | ^†^ | ^†^ | ^†^ | ^†^ | ^†^ |

The table provides a quick overview of the results of the performed linear mixed models using the total UPDRS-III score as outcome. On the left side, results for models using an asymmetry index (AI) of 0% as cutoff are shown, while the right side depicts results for an asymmetry index of 5%.

DaT = Dopamine Transporter, LA = less affected, LEDD =Levodopa Equivalent Daily Dose, MA = more affected, MoCA = Montreal Cognitive Assessment, MDS-UPDRS = Movement Disorder Society - Unified Parkinson’s Disease Rating Scale

*Based on separate models for more and less affected putamen and caudate SBRs, we applied Bonferroni correction for multiple comparisons. The adjusted alpha level was set at 0.00625 (0.05/8). Therefore, results with a p-value < 0.006 were considered significant. Striatal SBRs and mean values were not included in the multiple comparison adjustment, as they are encompassed within the analyses of the putamen and caudate.* ^†^*<.05, *<.006, **<0.001, ***<.0001*

**Supplementary Table 9** – Capability of putaminal DaT signals to monitor UPDRS-III score progression without tremor items, restricted to patients with aligned clinical and imaging dominant affected sides

| Linear mixed model analysis of putaminal dopamine transporter signal-dependent increase in motor symptom severity without tremors for | | | | |
| --- | --- | --- | --- | --- |
|  | Less affected putaminal signal AI = 0 | | Less affected  putaminal signal AI = 5% | |
| Predictors | ß | p-value  95% CI | ß | p-value  95% CI |
| Intercept | 3.06 | .32  -2.97:9.10 | 2.09 | .55  -4.76:8.93 |
| Fixed effects (time-DEPENDENT) | | | | |
| Linear time (Month) | 0.14 | <.001***  0.09:0.20 | 0.13 | <.001***  0.07:0.19 |
| putaminal  DaT signal | -2.03 | <.01**  -3.28:-0.78 | -2.48 | <.001***  -3.87:-1.09 |
| Interaction  (time*DaT Signal) | -0.06 | <.05*  -0.12:-0.00 | -0.06 | .08*  -0.12:0.01 |
| MoCA | -0.12 | .09  -0.26:0.02 | -0.08 | .33  -0.24:0.08 |
| LEDD | 0.20 | .75  -1.06:1.47 | 0.40 | .57  -1.00:1.80 |
| Depression medication (0 = no, 1 = SSRI, 2 = SNRI, 3 = serotonin modulating, 4 = others) | | | | |
| 1 | 1.99 | .07  -0.13:4.12 | 2.55 | <0.5*  0.27:4.83 |
| 2 | -0.45 | .84  -4.92:4.019 | 0.40 | .87  -4.45:5.25 |
| 3 | -2.15 | .33  -6.44:2.14 | -2.34 | .41  -7.95:3.27 |
| 4 | -8.47 | .09  -18.40:1.45 | -8.34 | .09  -18.04:1.37 |
| Fixed effects (time constant) | | | | |
| Age | 0.13 | <.001***  0.08:0.19 | 0.11 | <.001***  0.06:0.17 |
| Sex (0 = female, 1 = male) | | | | |
| 1 | 0.46 | .38; [-0.57:1.49] | 0.44 | .44  -0.68:1.56 |
| Handedness (0=right, 1=left, 2=mixed) | | | | |
| 1 | 0.93 | .28  -0.75:2.61 | 0.87 | .34  -0.94:2.69 |
| 2 | 0.33 | .85  -3.01:3.67 | -2.72 | .18  -6.69:1.25 |
| Disease duration | 0.02 | .18  -0.01:0.06 | 0.02 | .18  -0.01:0.06 |
| Educyrs | 0.07 | .40  -0.09:0.23 | 0.15 | .08  -0.02:0.33 |
| Random effects | SD | Variance | SD | Variance |
| Intercept (Time) | 4.17 | 17.37 | 3.95 | 15.57 |
| Slope (time) | 0.09 | 0.01 | 0.08 | 0.01 |
| Residual | 3.39 | 11.48 | 3.48 | 12.14 |

This table presents the results of two linear mixed models evaluating the potential effects of misalignments between the imaging-determined and clinically determined more affected sides. The clinically more affected side was defined as the side with higher UPDRS-III scores, while the imaging-determined more affected hemisphere was identified by calculating the asymmetry index and applying cutoffs of 0% and 5%, respectively. For consistency, two-sided p-values and confidence intervals (CI) are reported, although the strong a priori hypothesis would justify the use of a one-sided test for the interaction term.

*<.05, **<.01, ***<.001 ß = unstandardized coefficients, CI = Confidence Intervals, DaT = Dopamine Transporter, EDUCYRS = years of education, LEDD = Levodopa Equivalence Daily Dose, MoCA = Montreal Cognitive Assessment, SSRI = Selective Serotonin Reuptake Inhibitor, SNRI = Serotonin Norepinephrine Reuptake Inhibitor, UPDRS-III = Unified Parkinson’s Disease Rating Scale motor-part

**Supplementary Table 10** - Patient identifiers from the Parkinson’s Progression Marker Initiative (PPMI) database

| **Patient Identifiers** |
| --- |
| 3001, 3002, 3003, 3010, 3012, 3014, 3018, 3020, 3021, 3023, 3024, 3027, 3028, 3051, 3052, 3056, 3058, 3059, 3061, 3062, 3066, 3067, 3068, 3076, 3077, 3078, 3080, 3083, 3086, 3088, 3089, 3102, 3105, 3107, 3108, 3110, 3111, 3113, 3116, 3118, 3119, 3120, 3122, 3123, 3124, 3125, 3126, 3127, 3128, 3130, 3131, 3132, 3134, 3150, 3154, 3162, 3166, 3168, 3173, 3174, 3175, 3176, 3178, 3179, 3180, 3181, 3182, 3184, 3185, 3186, 3190, 3203, 3207, 3209, 3211, 3212, 3214, 3220, 3225, 3226, 3227, 3228, 3229, 3230, 3231, 3233, 3234, 3251, 3252, 3253, 3254, 3269, 3272, 3275, 3278, 3305, 3307, 3308, 3309, 3312, 3313, 3321, 3323, 3325, 3327, 3328, 3360, 3364, 3374, 3375, 3377, 3378, 3383, 3392, 3400, 3403, 3406, 3407, 3409, 3415, 3417, 3418, 3419, 3420, 3421, 3422, 3423, 3429, 3430, 3431, 3432, 3433, 3434, 3435, 3436, 3439, 3440, 3442, 3443, 3444, 3445, 3446, 3448, 3451, 3454, 3455, 3459, 3461, 3462, 3467, 3469, 3470, 3471, 3472, 3473, 3475, 3476, 3482, 3500, 3502, 3504, 3505, 3506, 3507, 3514, 3516, 3522, 3528, 3530, 3532, 3540, 3542, 3556, 3557, 3558, 3559, 3564, 3567, 3574, 3575, 3577, 3584, 3585, 3586, 3587, 3589, 3592, 3593, 3601, 3604, 3605, 3606, 3607, 3608, 3609, 3612, 3616, 3617, 3621, 3622, 3625, 3629, 3631, 3632, 3634, 3638, 3650, 3653, 3654, 3657, 3659, 3660, 3661, 3664, 3665, 3666, 3700, 3701, 3702, 3704, 3705, 3707, 3708, 3709, 3710, 3711, 3752, 3757, 3758, 3760, 3762, 3763, 3766, 3770, 3775, 3777, 3778, 3780, 3781, 3789, 3802, 3808, 3814, 3815, 3819, 3822, 3824, 3825, 3826, 3828, 3829, 3830, 3832, 3834, 3835, 3838, 3856, 3863, 3866, 3869, 3870, 3900, 3903, 3904, 3905, 3910, 3911, 3914, 3916, 3951, 3954, 3957, 3960, 3961, 3963, 3964, 3970, 4005, 4011, 4012, 4013, 4019, 4022, 4024, 4029, 4030, 4033, 4034, 4035, 4037, 4051, 4052, 4054, 4056, 4057, 4058, 4059, 4065, 4070, 4072, 4073, 4074, 4076, 4077, 4078, 4091, 4092, 4093, 4094, 4096, 4098, 4099, 4101, 4103, 4106, 4107, 4108, 4109, 4110, 4111, 4112, 4113, 4114, 4115, 4117, 4121, 4122, 4123, 4124, 4125, 4126, 4135, 5015, 40366, 40533, 40538, 40541, 40555, 40578, 40585, 40586, 40592, 40603, 40607, 40690, 40691, 40692, 40693, 40694, 40702, 40703, 40704, 40707, 40709, 40713, 40714, 40735, 40744, 40776, 40781, 40816, 41184, 41380, 41382, 41384, 41399, 41401, 41410, 41412, 41420, 41486, 41508, 41519, 41521, 41522, 41568, 41578, 41664, 41749, 41886, 41989, 42033, 42034, 42171, 42172, 42269, 43046, 50028, 50044, 50088, 50157, 50175, 50192, 50621, 50860, 50901, 51186, 51252, 51440, 51551, 51625, 51632, 51844, 51971, 52062, 52146, 52530, 53060, 53339, 53595, 53600, 53988, 54144, 54161, 54197, 54265, 54695, 55124, 55251, 55441, 55615, 55875, 56558, 56744, 57090, 57127, 57813, 57869, 57887, 58030, 58420, 58454, 58510, 58671, 58783, 59121, 59507, 59649, 59724, 70188, 70326, 70463, 70818, 71189, 71809, 71978, 72784, 72826, 73115, 73935, 74067, 75480, 75484, 75505, 75524, 75562, 100001, 100005, 100006, 100007, 100012, 100017, 100018, 100267, 100268, 100842, 100878, 100889, 100891, 100898, 100905, 100911, 100952, 100972, 101018, 101026, 101038, 101047, 101070, 101092, 101124, 101146, 101174, 101179, 101187, 101279, 101295, 101330, 101476, 101477, 101479, 101492, 101513, 101516, 101557, 101742, 101744, 101748, 101751, 101756, 101799, 101841, 102003, 102012, 102027, 102054, 102068, 102293, 102305, 102420, 102475, 102479, 102484, 102978, 103813, 103914, 105711, 105854, 106703, 110192, 110212, 110219, 110220, 110360, 111278, 111545, 112729, 112926, 113446, 113460, 114137, 114195, 114265, 114272, 114526, 114534, 114615, 115448, 115451, 115687, 115820, 116008, 116216, 116531, 116733, 116742, 118352, 120544, 121619, 122645, 123594, 124793, 127075, 128196, 129515, 130013, 130156, 130828, 131602, 132338, 133472, 133489, 133507, 134456, 134605, 135579, 135704, 135861, 136646, 136681, 137264, 137426, 137450, 137482, 137842, 137886, 139859, 139982, 140027, 140258, 140568, 140671, 140722, 140761, 140762, 141135, 141692, 141696, 142004, 142007, 142957, 143116, 143119, 143192, 144120, 144147, 145939, 145940, 147077, 147191, 147294, 147650, 148093, 148699, 148945, 149116, 149511, 149516, 149937, 149940, 150414, 150505, 151050, 151669, 152369, 152582, 152600, 152616, 153027, 153089, 153191, 156665, 157229, 157410, 158359, 160040, 160057, 160231, 161236, 161673, 162793, 162941, 162994, 163265, 163420, 163459, 163791, 164268, 164491, 164589, 164765, 165174, 165676, 167222, 167580, 167657, 168195, 168299, 170472, 170519, 170727, 171083, 171525, 172260, 172370, 172413, 173266, 173814, 174151, 174340, 174364, 174615, 174735, 174871, 175925, 177395, 179527, 179541, 179894, 181671, 181684, 181825, 181957, 182199, 182341, 182727, 182848, 182990, 184459, 186108, 187800, 188069, 188170, 189935, 190005, 190360, 190603, 192111, 194803, 194971, 195159, 201630, 203717, 204012, 209141, 210085, 211239, 211886, 211902, 212042, 212954, 213005, 213675, 214241, 214900, 216668, 216970, 218340, 219593, 230283, 235810, 237482, 238181, 238548, 239947, 243027, 245300, 245573, 245591 |
